# Application of artificial intelligence in non-invasive cardiovascular imaging for coronary artery disease: a systematic review and meta-analysis

**DOI:** 10.3389/fcvm.2025.1664183

**Published:** 2025-12-12

**Authors:** Baiyun Liu, Joana Reis, Ankur Sharma, Wei Wang

**Affiliations:** 1 Medical Affairs Radiology, Bayer Healthcare Co. Ltd., Shanghai, China; 2 Medical Affairs, Radiology, Bayer AG, Oslo, Norway; 3 Medical Affairs Radiology, Bayer Medical Care Inc., Pittsburgh, PA, United States; 4 Medical Affairs Radiology, Bayer Healthcare Company, Beijing, China

**Keywords:** artificial intelligence, non-invasive imaging modalities, cardiovascular imaging, coronary artery disease, systematic review

## Abstract

**Introduction:**

Coronary artery disease (CAD) remains a leading cause of death worldwide. While non-invasive imaging techniques are widely used for diagnosis, their interpretation can be time-consuming and subject to intra- and inter-observer variability. Artificial intelligence (AI), including machine learning and deep learning, offers potential advantages in improving diagnostic accuracy and efficiency by rapidly processing large imaging datasets.

**Methods:**

A systematic review was conducted to evaluate current evidence on AI applications in non-invasive CAD imaging. Searches were performed in PubMed, Embase, Web of Science, Engineering Index, and the Cochrane Library for studies published between 2018 and 2023. A total of 122 studies were included in the evidence map, and 9 studies assessing AI for detecting ≥50% coronary stenosis were selected for meta-analysis.

**Results:**

The pooled sensitivity and specificity for detecting stenosis were 0.94 and 0.69, respectively, at the patient level, and 0.81 and 0.88 at the vessel level. The area under the SROC curve was 0.83 (patient level) and 0.92 (vessel level), indicating good diagnostic performance. High heterogeneity was observed across studies.

**Discussion:**

These findings suggest that AI holds promise for enhancing the diagnostic process in CAD imaging. However, variability in methodologies and AI implementation underscores the need for standardization and further prospective validation.

## 1 Introduction

Coronary artery disease (CAD) remains the leading cause of morbidity and mortality worldwide, affecting 315 million people globally in 2022 (1) and placing a heavy burden on healthcare systems. Early and accurate diagnosis is essential for effective and timely treatment, which contributes to improved patient outcomes. Non-invasive imaging modalities, such as computed tomography (CT), coronary computed tomography angiography (CCTA), and cardiac magnetic resonance (CMR) are being used increasingly for the diagnosis of CAD, providing valuable anatomical and functional information on the extent of the disease (2, 3). Both the American College of Cardiology/American Heart Association (ACR/AHA) (4) and the European Society of Cardiology (5) endorse CCTA or functional imaging as initial tests for many symptomatic patients. However, the increasing use of non-invasive modalities also poses some challenges. Traditionally, the interpretation of radiological images has been undertaken by radiologists, which requires expertise, is often time consuming, and is limited by intra-observer and inter-observer variability (6). Moreover, with the increased use of non-invasive imaging modalities, the demand for and the workload of expert readers is on the rise (7).

**Figure 1:**
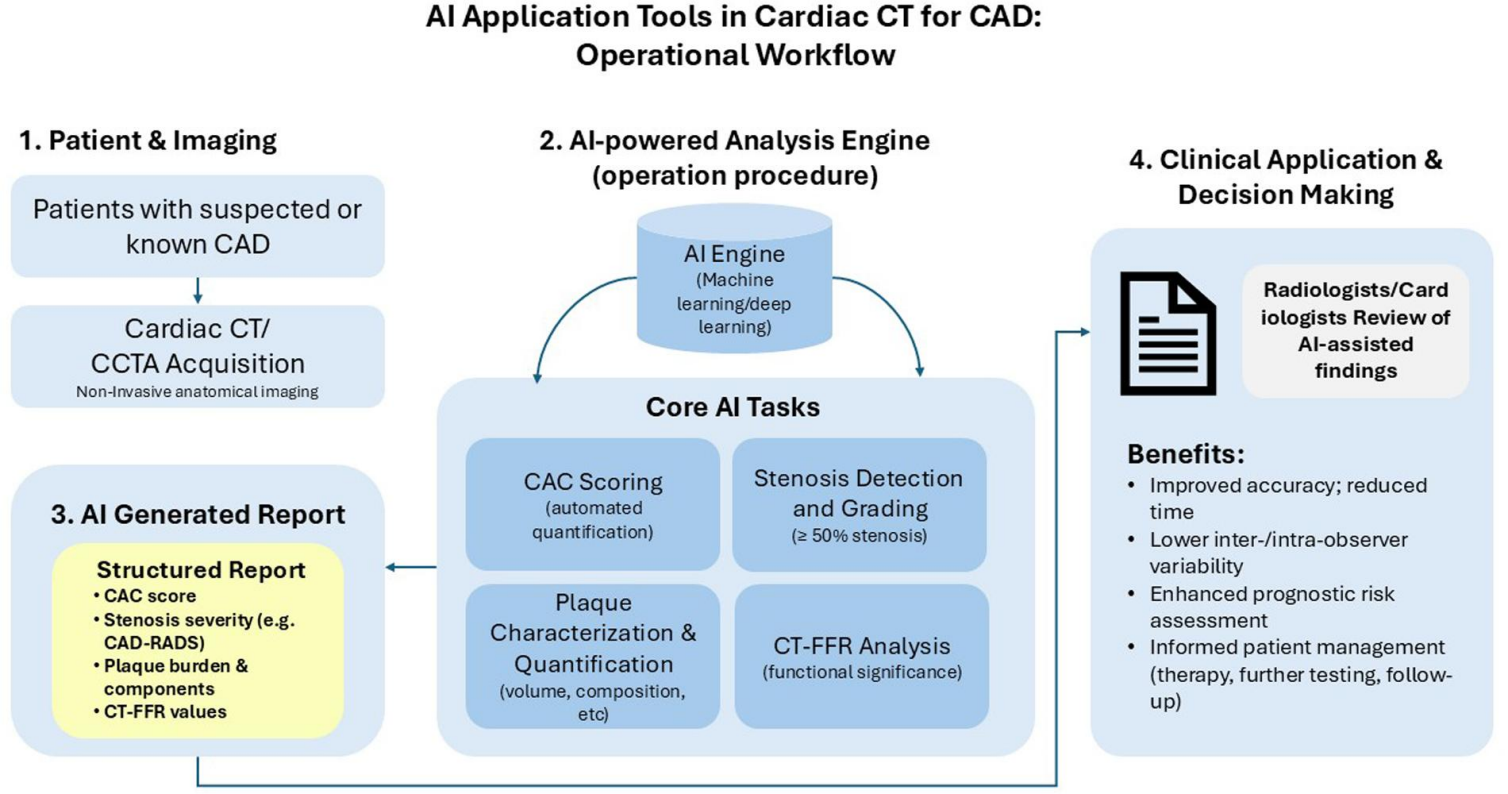
Workflow of artificial intelligence (AI) application tools in cardiac computed tomography (CT) for coronary artery disease (CAD) assessment. This diagram illustrates the operational procedure for implementing artificial intelligence in cardiac CT imaging for CAD evaluation. The workflow progresses sequentially from (1) patient imaging using cardiac CT/coronary computed tomography angiography (CCTA) modality, through (2) AI-powered analysis performing core tasks including coronary artery calcium (CAC) scoring, stenosis detection, plaque characterization, and CT-based fractional flow reserve (CT-FFR) analysis, to (3) the generation of a structured AI report including Coronary artery disease reporting and data system (CAD-RADS) classification, and finally (4) clinical application supporting informed patient management decisions. The AI system aims to improve diagnostic accuracy while reducing interpretation time and inter-observer variability.

The use of artificial intelligence (AI), including machine learning (ML) and deep learning (DL), could offer a solution by rapidly processing and analyzing large quantities of data, thereby reducing diagnosis time, and supporting the diagnostic decision-making process, resulting in improved diagnostic accuracy (6, 8). AI applications have demonstrated great promise in enhancing cardiovascular imaging, for example by automating coronary artery segmentation and scoring (9, 10), coronary artery calcification (CAC) scoring and risk assessment (11), coronary stenosis evaluation (12), coronary plaque segmentation (13), functional assessment of coronary stenosis (14), and by analyzing CMR images to derive clinically relevant measures (15) (Figure 1). AI has also been shown to be a useful tool for prognostic assessment, providing better risk assessment (16) and stratification (17). AI applications in cardiovascular imaging encompass a range of subtypes, including traditional ML algorithms, DL models such as convolutional neural networks (CNNs) and recurrent convolutional neural networks (RCNNs), as well as radiomics-based approaches. These techniques have been applied to automate coronary artery segmentation and scoring, evaluate stenosis, segment plaques, and perform functional assessments using imaging data.

Despite the promise of AI in cardiovascular imaging, several challenges remain, such as limited adoption of AI in clinical practice due a lack of cooperation and a knowledge gap between clinicians and data scientists, lack of high-quality standardized data, and difficulties with integration into existing workflows (18).

Nevertheless, there is increasing interest in the use of AI applications in cardiovascular imaging, which has led to a substantial increase in the number of publications on the subject. Systematic literature reviews and meta-analyses provide comprehensive, evidence-based insights by summarizing and pooling data from numerous studies, enabling informed decision-making, and in the context of AI, improving the reliability and fairness of AI apps, and ensuring better patient outcomes in clinical settings such as CAD. A systematic literature review can collect and synthesize research studies in the field, providing a systematic overview of the current state of research in the area. A meta-analysis goes beyond a systematic literature review and combines results from multiple research studies using statistical methods to critically evaluate diagnostic performance in specific clinical settings across various algorithms. It serves as a foundation for improving AI applications, ensuring their clinical relevance, and guiding their adoption in cardiovascular care. We undertook a systematic review and meta-analysis to evaluate existing evidence on the application of AI to non-invasive CAD imaging, focusing on 5 years of advancement (2018–2024), and assessed the diagnostic performance of AI applications in CAD imaging.

## 2 Methods

### 2.1 Search strategy

A comprehensive literature search was performed in 5 key databases—PubMed, Embase, Web of Science, Engineering Index, and the Cochrane Library—for articles published between January 2018 and September 2023. The selection of these databases was intentional to ensure broad coverage of this interdisciplinary topic. PubMed and Embase were chosen for their extensive biomedical and clinical content. Web of Science was included for its multidisciplinary scope, while the Engineering Index was searched to capture relevant publications from the computer science and engineering fields where AI technologies are developed. Finally, the Cochrane Library was searched to identify any existing systematic reviews or registered trials on this topic. Complete search strategies are included in the online supplement.

### 2.2 Study selection

Title and abstract screens were performed independently by two reviewers to minimize selection bias. Following this initial screen, full-text articles of all potentially relevant studies were retrieved and assessed for eligibility by the same two reviewers. Inter-rater reliability was periodically assessed and confirmed to be high throughout the screening process. Any disagreements at either the title/abstract or full-text screening stage were resolved through consensus discussions. If consensus could not be reached, a third reviewer was available for adjudication.

### 2.3 Inclusion and exclusion criteria for evidence mapping

Studies were selected for evidence mapping if they were: performed in adults diagnosed with or suspected of CAD (such as angina pectoris, coronary stenosis, myocardial infarction, coronary artery atherosclerosis, and coronary artery vulnerable plaque); included patients who underwent cardiovascular imaging, especially computerized tomography fractional flow reserve (CT-FFR), CMR, or imaging for CAC or plaques or stenosis; were retrospective or prospective in design; applied AI to non-invasive cardiovascular imaging modalities (including CMR, CCTA, and CT); and included diagnosis or prognosis/prediction of CAD as study outcomes.

Studies were excluded if they: involved patients with cardiovascular diseases who were <18 years of age; included patients who had a prior history of cardiovascular surgery; did not apply AI; did not involve patients undergoing cardiovascular imaging, especially CT-FFR, CMR, or imaging for CAC or plaques or stenosis; were based on non-radiological (such as electrocardiogram, ultrasound, and optical coherence tomography) or invasive imaging (modalities such as digital subtraction angiography and intravascular ultrasound); or were case reports or case series.

Our aim was to focus specifically on the application of AI to major non-invasive *radiological* modalities like CT and CMR, as they represent a distinct and rapidly advancing field in CAD diagnosis. Non-radiological data sources (e.g., electrocardiogram) or other imaging modalities that fall outside the defined scope of this review (e.g., ultrasound, optical coherence tomography) were excluded. This criterion was established to maintain a clear focus on AI applications in CT and CMR imaging and to avoid methodological heterogeneity that arises from fundamentally different imaging principles and data types. Similarly, studies centered on invasive imaging (such as digital subtraction angiography and intravascular ultrasound) were excluded, although their use as a reference standard for comparison was permitted.

### 2.4 Inclusion and exclusion criteria for the meta-analysis

Studies were selected for the meta-analysis if they: involved adults diagnosed with or were suspected of CAD; were prospective or retrospective in design; applied AI to non-invasive cardiovascular imaging modalities (CMR, CCTA, and CT); used invasive coronary angiography (ICA) as a reference standard; and assessed the diagnostic performance of AI when detecting ≥50% coronary stenosis at the patient and vessel level. Diagnostic performance was measured by accuracy, sensitivity, specificity, positive predictive value, negative predictive value, area under the receiver operating characteristic (ROC) curve, true positive, false positive, true negative, and false negative.

Studies were excluded based on the criteria used during selection for evidence mapping and if the studies did not assess plaques or stenosis.

### 2.5 Data extraction

Data were extracted by one reviewer and checked by another reviewer by using a standardized data extraction form. Any disagreements were resolved by discussion and, if necessary, by adjudication by a third reviewer. Data extracted for evidence mapping included information about the studies (first author, year of publication, country), study design (prospective/retrospective), participants (indication, sample size), outcomes, (diagnosis, prognosis/prediction), and interventions/controls (AI type, modality).

Data extracted for the meta-analysis included information about the studies (first author, year of publication, country), study design (prospective/retrospective, index test, reference standard), participants (sample size, indication), interventions/controls (AI type, modality), and outcomes (specific outcome of diagnosis or prognosis/prediction, definition of outcome, performance measurements).

### 2.6 Statistical analysis

Evidence mapping included descriptive and visual representations of data (bar charts, pie charts, and line graphs) to represent the regional distribution of the included studies, the distribution of different cardiovascular imaging modalities in combination with AI, and the distribution of CT imaging modalities using AI.

The meta-analysis was performed using the MIDAS module of Stata SE version 15.1 software and a random-effects model. This module was specifically chosen because it is designed for the meta-analysis of diagnostic test accuracy and implements the recommended bivariate random-effects model. This approach is superior for our analysis as it jointly models sensitivity and specificity, accounting for the inherent correlation between these two metrics. Furthermore, MIDAS facilitates the direct calculation of pooled sensitivity, specificity, likelihood ratios, and diagnostic odds ratios, as well as the generation of summary receiver operating characteristic (SROC) curves, all of which were essential for our evaluation.

Only studies that directly reported or allowed for the calculation of true positive, false positive, true negative, and false negative, and were conducted using patient- and vessel-level data separately were included in the meta-analysis. Diagnostic accuracy was assessed using pooled analyses of sensitivity, specificity, positive likelihood ratio, negative likelihood ratio, and diagnostic odds ratio with corresponding 95% confidence intervals (CIs). A summary ROC (SROC) curve was constructed using a bivariate regression method to identify anomalous checks that resulted in the expected trade-off between sensitivity and specificity. Area under the curve (AUC) was used to summarize the test's inherent ability to distinguish between detecting ≥50% stenosis and <50% stenosis, and a *p*-value of <0.05 was considered statistically significant.

Heterogeneity was assessed using the *I*^2^ statistic, with *I*^2^ values interpreted as follows: *I*^2^ of ≤25% indicates no heterogeneity, *I*^2^ of 26% to 50% suggests a low degree of heterogeneity, *I*^2^ of 51%–75% indicates a moderate degree of heterogeneity, and *I*^2^ of ≥75% signifies a high degree of heterogeneity.

For the meta-analysis, risk of bias was assessed with QUADAS-2 (Quality Assessment of Diagnostic Accuracy Studies), a tool developed to assess the risk of bias in diagnostic test accuracy studies. The main source of bias included patient selection, index tests (the AI algorithm), reference standard, and flow and timing (19). Publication bias was assessed by Deek's funnel plots of patient- and vessel-level data.

## 3 Results

### 3.1 Included studies

**Figure 2:**
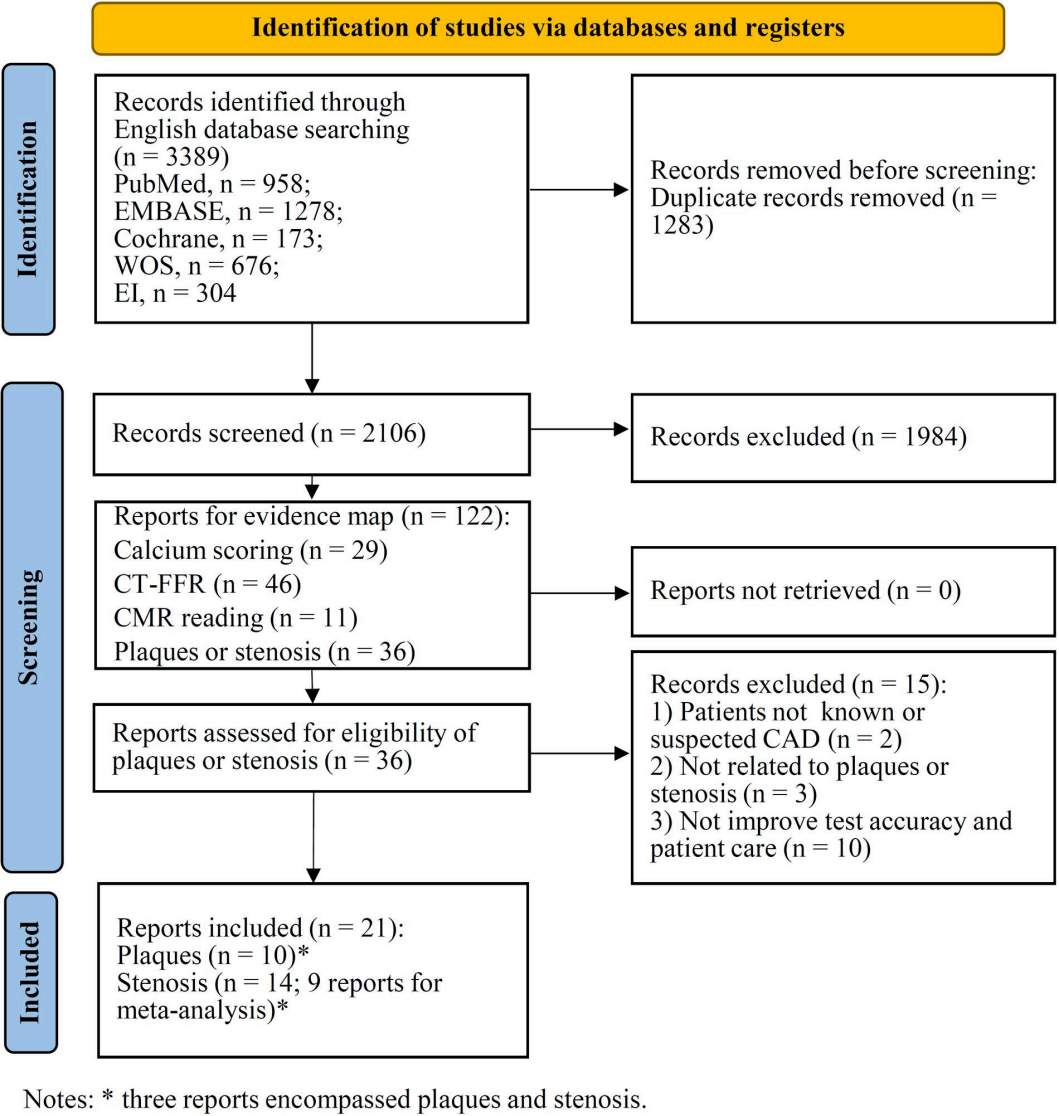
PRISMA flow diagram for study selection.

In total 3,389 records were retrieved from five databases (Figure 2). After eliminating duplicates, a total of 2,106 records were identified, of which 1,984 were excluded after title and abstract screening. A total of 122 studies were selected for evidence mapping (Supplementary Table 1), of which 46 were CT-FFR studies, 36 were plaque or stenosis studies, 29 were calcium scoring studies, and 11 were CMR studies. Subsequently, the full-text articles of the 36 plaque or stenosis studies, including 9 plaque studies, 16 stenosis studies, and 11 studies that imaged both plaques and stenosis, were screened for meta-analysis. Of the 16 stenosis studies, 9 studies (8 using patient-level and 6 studies using vessel-level data) were eligible for a meta-analysis assessing the diagnostic performance of AI when detecting ≥50% stenosis and reported or allowed for the calculation of true positive, false positive, true negative, and false negative data.

### 3.2 Evidence mapping

#### 3.2.1 Regional distribution of included studies

**Figure 3:**
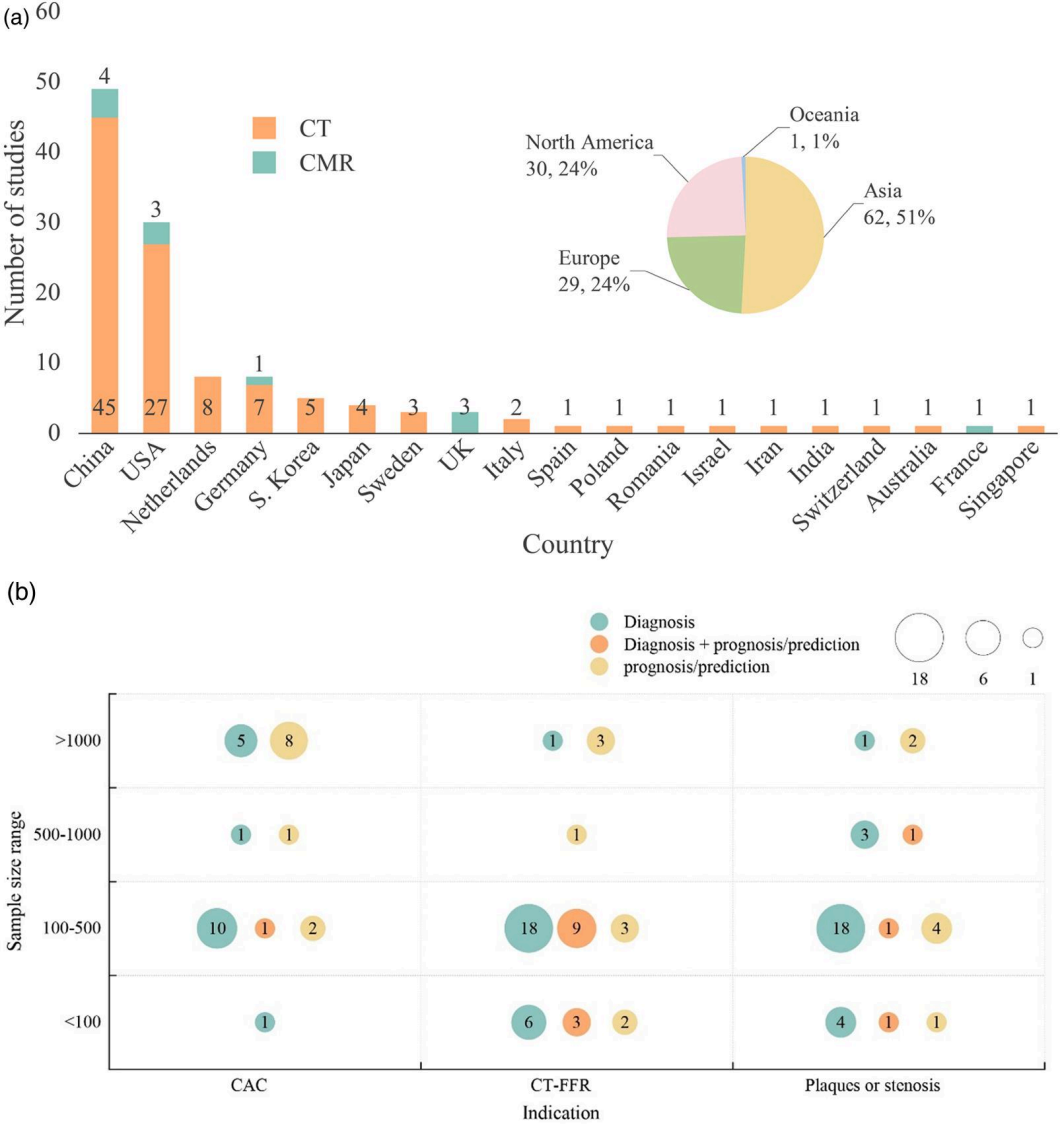
**(a)** Geographic distribution of evidence mapping studies (*n* = 122). Different colors represent geographical regions and modalities. **(b)** Type and sample size of evidence mapping studies using computed tomography modalities. Different colors represent study outcomes, with diagnostic studies indicated in green, prognostic/predictive studies in yellow, and both diagnostic and prognostic/predictive studies in orange.

The geographic distribution of 122 studies selected for evidence mapping is shown in Figure 3A; Table 1. Studies were conducted mostly in Asia (*n* = 62; 50.8%), followed by North America (*n* = 30; 24.6%) and Europe (*n* = 29; 23.8%), with more studies conducted in China (*n* = 49) and the USA (*n* = 30) than in other countries. Among the 49 studies conducted in China, 45 were CT, and 4 were CMR studies. In the other Asian countries and Oceania, 5 studies were conducted in South Korea (all CT studies), 4 were conducted in Japan (all CT studies), and 1 study each was conducted in Australia, Iran, Israel and Singapore (all CT studies). The studies in North America included 30 studies, all conducted in the USA (27 CT and 3 CMR studies). Studies conducted in the European region included 8 studies in the Netherlands (all CT), 8 in Germany (7 CT and 1 CMR), 3 in the UK (all CMR), 3 in Sweden (all CT), 2 in Italy (both CT), and 1 each in Switzerland (CT), Poland (CT), Romania (CT), Spain (CT), and France (CMR).

**Table 1:** Key characteristics of the evidence mapping studies (*n* = 122).

Characteristics	Studies, *n* (%)
Clinical Indications	
Coronary artery calcium scoring	29 (23.6)
Coronary artery stenosis or atherosclerosis	36 (29.5)
CT-FFR	46 (37.4)
Cardiac MRI reading	11 (8.9)
Year of publication	
2023	24 (19.5)
2022	40 (32.5)
2021	15 (12.2)
2020	22 (17.9)
2019	12 (9.8)
2018	10 (8.1)
Country of author	
China	49 (39.8)
USA	30 (24.6)
Germany	8 (6.5)
Netherlands	8 (6.5)
South Korea	5 (4.1)
Japan	4 (3.3)
Sweden	3 (2.4)
United Kingdom	3 (2.4)
Italy	2 (1.6)
Australia	1 (0.8)
France	1 (0.8)
India	1 (0.8)
Iran	1 (0.8)
Israel	1 (0.8)
Poland	1 (0.8)
Romania	1 (0.8)
Singapore	1 (0.8)
Spain	1 (0.8)
Switzerland	1 (0.8)
Sample size of the study (no. of patients)	
≤100	19 (15.6)
100–500	68 (55.7)
500–1,000	8 (6.5)
>1,000	21 (17.1)
NA	6 (4.9)
Type of study (based on the outcome)	
Diagnostic	75 (61.5)
Prognostic/Predictive	31 (25.4)
Both	16 (13.1)

CT-FFR, computerized tomography fractional flow reserve; MRI, magnetic resonance imaging.

#### 3.2.2 Distribution of CT and CMR imaging modalities using AI applications

Among the included studies that used CT imaging modalities (*n* = 111), AI-based analysis was used in 46 CT-FFR, 36 plaque or stenosis, and 29 calcium scoring studies. Figure 3B presents evidence mapping results categorized by imaging indications related to CAD (including CAC, CT-FFR, and plaques or stenosis) and sample size (>1,000, 500–1,000, 100–500, <100 patients). More than half the studies (*n* = 62; 55.7%) had sample sizes of 100–500 patients.

Among the 46 CT-FFR studies, 24 were diagnostic, 9 were prognostic/predictive, and 12 studies were both. The 36 plaque or stenosis studies included 26 diagnostic studies, 7 prognostic/predictive studies, and 3 studies were both diagnostic and prognostic/predictive. Of the 29 calcium scoring studies, 17 were diagnostic, 11 were prognostic/predictive, and one study was both.

Of the 11 studies that used CMR imaging modalities, 8 were diagnostic studies and 3 were prognostic/predictive studies.

### 3.3 Meta-analysis of studies using AI applications for detecting ≥50% stenosis

The 9 studies eligible for meta-analysis on the application of AI for detecting ≥50% stenosis included a total of 2,263 patients (Table 2) (13, 20–27). Of these studies, 5 reported both patient-level and vessel-level data, 3 reported only patient-level data, and 1 reported vessel-level data, resulting in 8 studies for meta-analysis of patient-level (13, 20, 21, 23–27) and 6 studies for meta-analysis of vessel-level data (13, 22–26). Seven of the studies were conducted in China and 2 in the USA, with 3 studies each being published in 2023 and 2022, two studies in 2021, and one study in 2020. All studies used AI for CCTA, including DL in three studies, CNN in three studies and ML/AI-guided quantitative CT in one study (i.e., one study used both DL and ML). The reference standard for all studies was ICA.

**Table 2:** Characteristics of stenosis studies included in the meta-analysis (*n* = 9).

ID	Study	Country	Study Design	Sample Size	AI Algorithm	Performance in detecting ≥ 50% stenosis
95	Huang et al. (2023) (20)	China	Retrospective/Single center	346 patients	AI system (Shukun tech)	(**Patient level**) AUC: 0.83; Acc:0.82; Sens: 0.79; Spec: 0.87; PPV: 0.89; NPV: 0.74
97	Lipkin et al. (2022) (21)	USA	Retrospective *post-hoc* of CREDENCE trial	301 patients	AI system (Cleerly)	(**Patient level**) AUC: 0.88; Sens: 0.95; Spec: 0.63; PPV: 0.75; NPV: 0.92
98	Han et al. (2020) (22)	China	Retrospective/Single center	50 patients/68 vessels	AI system (Shukun tech)	(**Vessel level**) AUC: 0.87; Acc:0.86; Sens: 0.88; Spec: 0.85; PPV: 0.73; NPV: 0.94
99	Liu et al. 2021 (23)	China	Retrospective/Single center	165 patients/680 vessels	AI system (Shukun tech)	(**Patient level**) AUC: 0.90; Acc: 0.90; Sens: 0.91; Spec: 0.82; PPV: 0.98; NPV: 0.50 (**Vessel level**) AUC: 0.90; Acc:0.89; Sens: 0.81; Spec: 0.94; PPV: 0.89; NPV: 0.90
102	Lin et al. (2022) (13)	USA	Retrospective/Multicenter	50 patients/150 vessels	ConvLSTM network	(**Patient level**) Acc: 0.90; Sens: 1.00; Spec: 0.68; PPV: 0.87; NPV: 1.00 (**Vessel level**) Acc:0.93; Sens: 0.98; Spec: 0.91; PPV: 0.84; NPV: 0.99
103	Xu et al. (2021) (24)	China	Retrospective/Multicenter	527 patients/2073 vessels	AI system (Shukun tech)	(**Patient level**) AUC: 0.81; Sens: 0.90; Spec: 0.55; PPV: 0.91; NPV: 0.52 (**Vessel level**) AUC: 0.83; Sens: 0.66; Spec: 0.86; PPV: 0.76; NPV: 0.79
104	Xu et al. (2022) (25)	China	Retrospective/Single center	306 patients/1224 vessels	AI system (Shukun tech)	(**Patient level**) AUC: 0.72; Acc:0.81; Sens: 0.86; Spec: 0.58; PPV: 0.90; NPV: 0.49 (**Vessel level**) AUC: 0.76; Acc:0.80; Sens: 0.67; Spec: 0.85; PPV: 0.68; NPV: 0.85
120	Han et al. (2023) (26)	China	Retrospective/Single center	200 patients/771 vessels	AI system (Shukun tech)	(**Patient level**) AUC: 0.80; Acc:0.80; Sens: 0.93; Spec: 0.59; PPV: 0.85; NPV: 0.78 (**Vessel level**) AUC: 0.84; Acc:0.84; Sens: 0.77; Spec: 0.83; PPV: 0.66; NPV: 0.89
121	Han et al. (2023) (27)	China	Retrospective/Multicenter	318 patients	AI system (Shukun tech)	(**Patient level**) AUC: 0.85; Acc:0.80; Sens: 0.90; Spec: 0.71; PPV: 0.95; NPV: 0.53

AI, artificial intelligence; Acc, accuracy; AUC, area under the receiver operating characteristic curve; NPV, negative predictive value; NR, not reported; PPV, positive predictive value; RCNN, region-based convolutional neural networks; Sens, sensitivity; Spec, specificity.

#### 3.3.1 Analysis of AI diagnostic performance from patient-level data

**Figure 4:**
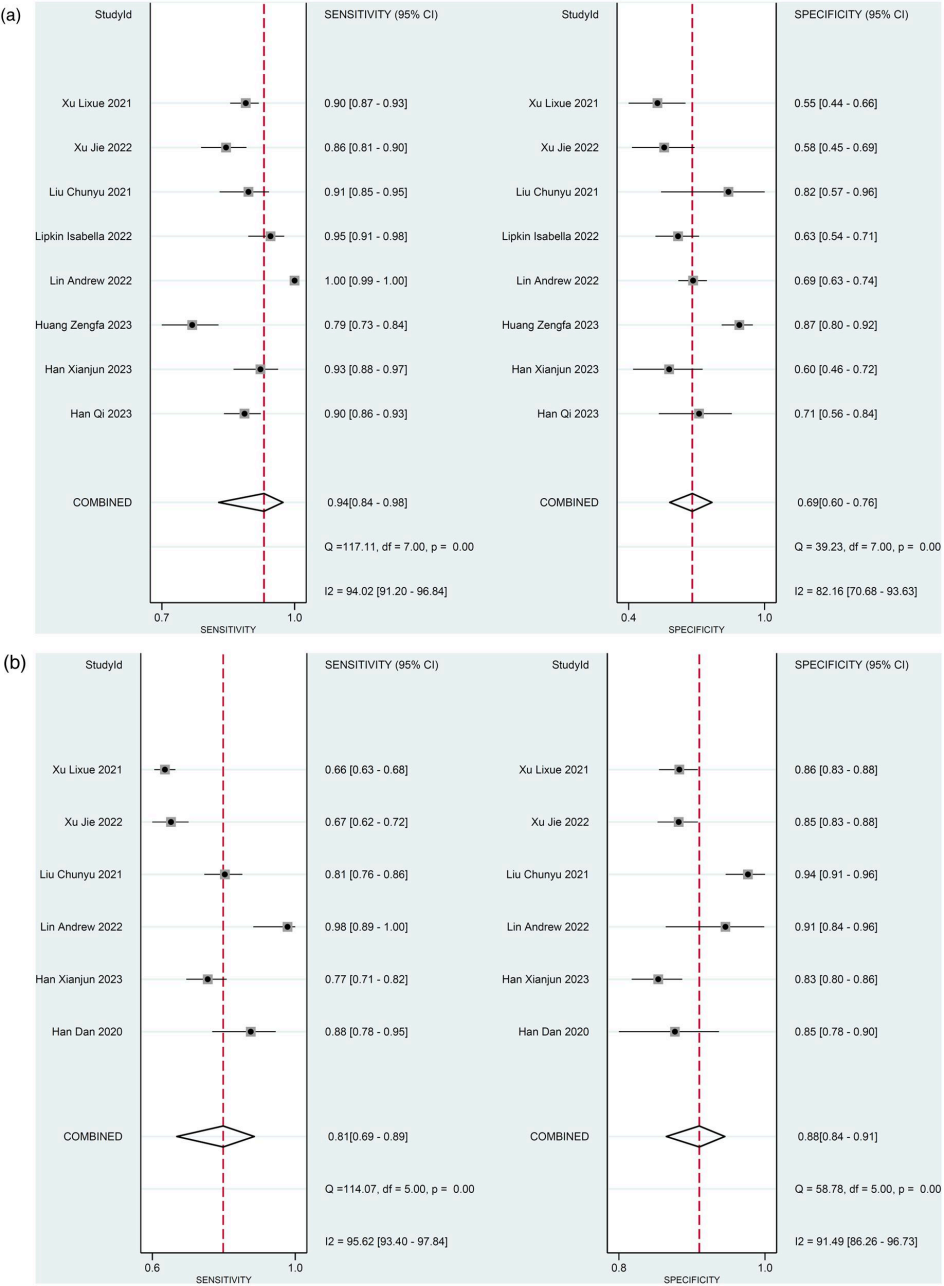
Diagnostic performance of AI to detect ≥50% stenosis. Forest plots of sensitivity and specificity **(a)** using patient-level data and **(b)** vessel-level data.

**Figure 5:**
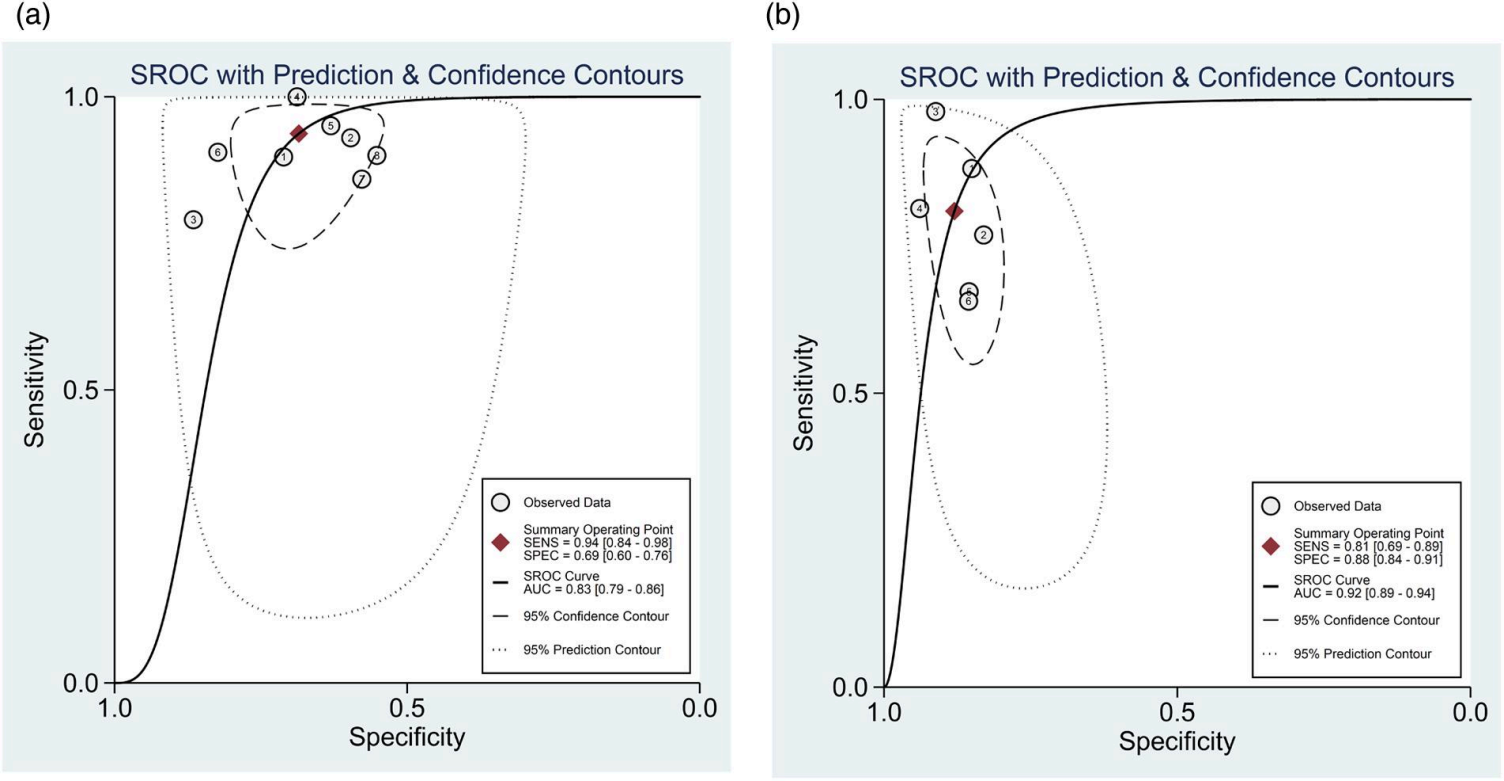
Summary receiver operating characteristic curve of the diagnostic performance of AI in detecting ≥50% stenosis using **(a)** patient-level and **(b)** vessel-level data. Each circle indicates one included study.

For the 8 studies evaluating the diagnostic performance of AI for detecting ≥50% stenosis using patient-level data (13, 20, 21, 23–27), the meta-analysis calculated the pooled sensitivity to be 0.94 (95% CI 0.84–0.98) and the pooled specificity to be 0.69 (95% CI 0.60–0.76) (Figure 4A). The pooled positive likelihood, negative likelihood, and diagnostic odds ratios were 2.98 (95% CI 2.30–3.85), 0.09 (95% CI 0.04–0.23), and 32.5 (95% CI 11.63–90.85), respectively (Supplementary Figure 1). A likelihood ratio scatter plot illustrates the summary points of positive and negative likelihood ratios for the 8 studies (Supplementary Figure 2a). The area under the SROC curve was 0.83 (95% CI 0.79–0.86) (Figure 5A), suggesting very good diagnostic performance. Using a pre-test probability of 50%, the post-test probability of detecting ≥50% stenosis was 75% (Fagan's nomogram shown in Supplementary Figure 3a). However, heterogeneity was observed between studies, with *I*^2^ values exceeding 60% for sensitivity, specificity, likelihood ratio, and diagnostic odds ratio (Figure 4B; Supplementary Figure 1).

#### 3.3.2 Analysis of AI diagnostic performance from vessel-level data

For the 6 studies evaluating the diagnostic performance of AI for detecting ≥50% stenosis using vessel-level data (13, 22–26), the meta-analysis calculated the pooled sensitivity to be 0.81 (95% CI 0.69–0.89) and the pooled specificity to be 0.88 (95% CI 0.84–0.91) (Figure 4B). The pooled positive likelihood, negative likelihood, and diagnostic odds ratios were 6.74 (95% CI 4.68–9.72), 0.22 (95% CI 0.12–0.38), and 31.21 (95% CI 13.26–73.47), respectively (Supplementary Figure 4). A likelihood ratio scatter plot illustrates the summary points of positive and negative likelihood ratios for the 6 studies (Supplementary Figure 2b). The area under the SROC curve was 0.92 (95% CI 0.89–0.94) (Figure 5B), and using a pre-test probability of 50%, the post-test probability of detecting ≥50% stenosis was 87% (Fagan's nomogram shown in Supplementary Figure 3b). Heterogeneity was observed, with *I*^2^ values exceeding 90% for sensitivity, specificity, likelihood ratio, and diagnostic odds ratio (Figure 4B; Supplementary Figure 4).

#### 3.3.3 Risk of bias and publication bias

**Figure 6:**
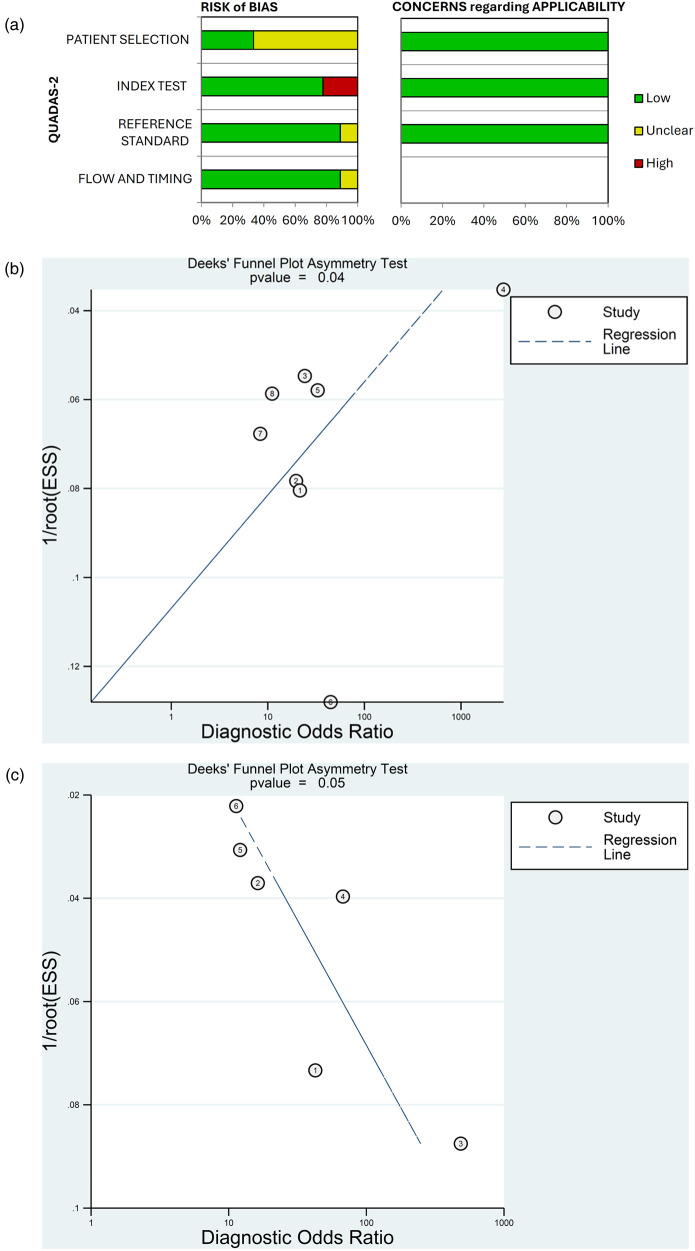
Quality assessment of diagnostic accuracy studies using QUADAS-2 **(a)** and publication bias assessed by Deek's funnel plots for **(b)** patient-level and **(c)** vessel-level data.

The risk of bias in diagnostic accuracy for the 9 studies included in the meta-analysis is presented in Figure 6A. Overall, the risk of bias on QUADAS-2 was unclear for the patient selection domain, and low for the index test, reference standard, and flow and timing domains. In terms of individual studies, in the patient-selection domain, 3 studies had low risk and 6 had unclear risk of bias (Figure 6A; Supplementary Table 2). In the index domain, 2 studies had high risk and 7 had low risk of bias. For the reference and flow and timing domains, no studies had high risk, 8 studies in each domain had low risk and 1 study in each domain had unclear risk of bias. Concern regarding applicability was low for all studies in the patient selection, index test and reference standard domains.

Deek's funnel plots found a low probability of publication bias for the studies included in the meta-analysis for patient-level (*p* = 0.04) and vessel-level data (*p* = 0.05) (Figures 6B,C).

### 3.4 Plaque imaging studies

A total of 10 plaque imaging studies were selected involving 2,822 participants (Table 3). Of these, 6 studies were diagnostic, 2 were prognostic/predictive, and 2 were both diagnostic and prognostic/predictive studies. All studies used AI for CCTA, including ML in 5 studies and CNN in 4 studies. The studies varied in how AI was used to quantify plaques (Table 3), and no meta-analysis was conducted.

**Table 3:** Characteristics of plaque studies (*n* = 10).

ID	Study	Sample Size	Country	AI Application	Modality	Objective	Results	Performance measurements	Index Test	Reference standard
94	Jin et al. (2022) (28)	505 patients	China	CNN + Radiomics based ML	CCTA	Coronary plaque detection and classification	The automatic workflow was proposed to detect and analyze coronary plaques with high accuracy and efficiency, showing the potential in clinical application	Sens: 0.83; Spec: 0.91; PPV: 0.83; NPV: 0.91; AUC: 0.87; Acc: 0.87; ICC: > 0.9; Processing time: 56.2 s; F value: > 5	NR	Expert reader
98	Han et al. (2020) (22)	150 patients	China	CNN	CCTA	Coronary plaque classification (calcified plaque, partially calcified plaque and noncalcified plaque)	The proposed CCTA-AI is relatively accurate in analyzing plaque features compared to traditional CCTA	Sens: 0.88; Spec: 0.85; PPV: 0.73; NPV: 0.94; Acc: 0.86; AUC: 0.87	NR	Traditional CCTA
102	Lin et al. 2022 (13)	921 patients	International, multicenter	DL	CCTA	Measure coronary plaque volume and segment coronary plaque (total plaque, calcified plaque and noncalcified plaque)	Deep learning system provides rapid measurements of plaque volume from CCTA that agree closely with expert readers and intravascular ultrasound, and could have prognostic value for future myocardial infarction	ICC of total plaque volume: 0.95; ICC of minimal luminal area 0.90	Expert/IVUS	NR
92	Yunus et al. (2022) (29)	202 patients	Malaysia	Radiomics based ML	CCTA	Atherosclerotic plaques classifications (normal, calcified, mixed, or non-calcified)	Auto-WEKA showed promising results in obtaining the best classifier among 39 machine learning for the classification of the calcified plaques compared to normal, non-calcified, and mixed plaques based on a CCTA-based radiomic dataset	Sens: 0.73; Spec: 0.91; PPV: 0.65; NPV: 0.94; Acc: 0.87; AUC: 0.928	NR	Radiologist report
107	Li et al. (2022) (30)	36 patients/350 plaques	China	Radiomics based ML	CCTA	Identification of vulnerable coronary plaques	Radiomics-based ML models showed better diagnostic ability than the conventional CCTA features at assessing coronary plaque vulnerability.	Sens: 0.88; Spec: 0.81; AUC: 0.90; Acc: 0.85; ICC: 0.983; Diagnostic ability: 0.78	Conventional CCTA	Pathology
116	Lin, Kolossvary, et al. (2022) (31)	120 patients	Australia	Radiomics based ML	CCTA	Culprit coronary lesions discrimination	Culprit lesions and highest-grade stenosis nonculprit lesions in MI have distinct radiomic signatures compared with lesions in stable CAD. Within the vulnerable patient may exist individual vulnerable plaques identifiable by CCTA-based precision phenotyping	AUC for the addition of quantitative plaque parameters to HRP: 0.76 AUC for addition of radiomic features to quantitative plaque parameters and HRP: 0.86	Lesions in Stable CAD	NR
108	Tesche et al. (2021) (32)	361 patients	Germany	ML	CCTA	Long-term prediction of MACE	Integration of a ML model improves the long-term prediction of MACE when compared with conventional CT risk scores, adverse plaque measures, and clinical information	AUC: 0.96	Conventional CT risk score	NR
110	Jonas et al. (2022) (33)	232 patents	USA	DL (AI-QCT)	CCTA	Coronary plaque and plaque components quantification	High variability remains among readers with high discordance compared to AI-QCT in quantifying specific high risk coronary plaque components	Spearman coefficients for correlation among expert readers 1, 2, and 3: 0.362, 0.353, and 0.442, respectively; Weighted kappa coefficients for agreement between AI and readers 1, 2, and 3: 0.224, 0.261, 0.166, respectively	Readers	NR
112	Li et al. (2023) (34)	132 patients/240 lesions	China	ML	CCTA	ACS prediction	ML model combining plaque characteristics, hemodynamic parameters and PCAT attenuation performed best in predicting the culprit lesion	(Logistic regression model) Sens: 0.89; Spec:0.59; Acc: 0.72; PPV: 0.62; NPV: 0.88. (Prediction performance of model 5) AUC: 0.819	Model with different predictors	NR
87	Zreik et al. (2018) (35)	163 patients	Netherlands	RCNN	CCTA	Detect (plaque of any type vs. no plaque) and classify (no plaque, non-calcified, mixed, calcified)	Automatic detection and classification of coronary artery plaque and stenosis are feasible, enabling automated triage of patients to those without coronary plaque and those with coronary plaque in need for further cardiovascular workup	Accuracy: LAD 0.78, LCx 0.81, RCA 0.72 F1 score: LAD 0.69, LCx 0.59, RCA 0.52 Cohen's kappa coefficient (*κ*): LAD 0.65, LCX 0.62, RCA 0.53	NR	Expert reader

AI-QCT, artificial intelligence-guided quantitative computed tomography; AUC, area under the receiver operating characteristic curve; CAD, coronary artery disease; CNN, convolutional neural network; CT, computed tomography; CTA, computed tomography angiography; CCTA, coronary computed tomography angiography; CCTA-AI, CCTA-artificial intelligence; DL, deep learning; ICC, intraclass correlation coefficient; LAD, left anterior descending artery; LCx, left circumflex artery; MACE, major adverse cardiovascular events; ML, machine learning; MI, myocardial infarction; NPV, negative predictive value; NR, not reported; PCAT, pericoronary adipose tissue; PPV, positive predictive value; RCA, right coronary artery; RCNN, region-based convolutional neural networks; Sens, sensitivity; Spec, specificity.

## 4 Discussion

This systematic review and meta-analysis evaluated existing evidence on the application of AI to non-invasive CAD imaging and the diagnostic performance of AI applications in CAD imaging. To our knowledge, this is the first meta-analysis to assess the diagnostic performance of AI applications for detecting ≥50% stenosis. We found 122 studies that used AI applications in non-invasive cardiovascular imaging of patients with known or suspected CAD, of which approximately half (51%) were conducted in Asia, and more studies were undertaken in China and the USA than in other countries. Our evidence mapping identified 111 studies utilizing CT-based techniques, compared to only 11 focused on CMR. This skew is not due to our search strategy, but rather indicates that the bulk of recent AI research in non-invasive CAD imaging has been concentrated on CT. Several factors likely contribute to this imbalance. First, CCTA is a frontline modality for the anatomical assessment of CAD, making large, retrospective datasets widely available for training AI models. Second, CT data, which is based on standardized Hounsfield units, is inherently more uniform than CMR data, where signal intensity can vary significantly depending on sequence parameters, hardware, and field strength. This data standardization simplifies the development and validation of AI algorithms across different sites. Consequently, while AI shows great promise for CMR in applications like tissue characterization and flow quantification, the evidence base for its diagnostic use in CAD is less mature. The studies identified ranged in sample sizes from <100 to >1,000 patients, with more than half (55.7%) of the studies having sample sizes of 100–500 patients. The distribution of studies across different sample sizes indicates a need for larger, more comprehensive studies to validate their findings and improve the generalizability of results. Studies with sample sizes of <100 patients highlight emerging areas that require further studies.

Among the 46 AI-based CT-FFR studies, we found 12 studies related to both diagnosis and prognosis/prediction, suggesting significant advancement in non-invasive evaluation of CAD that can effectively assess both immediate and long-term patient outcomes. More than half of the CT-FFR studies (30 studies) had moderate sample sizes of between 100 and 500 patients. These results indicate a balanced approach to addressing both diagnostic and prognostic/predictive needs and a focus on larger datasets.

We found 36 studies focusing on plaques or stenosis, which shows that there is growing interest in understanding how AI can analyze imaging characteristics of plaques and stenosis to predict patient outcomes. Indeed, studies of prognosis/prediction revealed that AI can effectively identify and characterize coronary plaques and stenosis, and can be used for assessing the risk of future acute coronary events (13, 32, 34).

Our review identified 29 studies focused on AI for CAC scoring, underscoring its established and evolving role in cardiovascular risk assessment. A deeper analysis reveals that AI serves two primary functions in this domain: automated quantification and advanced risk stratification. First, AI enables automated quantification, which improves workflow efficiency and consistency. This is particularly valuable for large-scale opportunistic screening, where deep learning models can automatically detect and score calcium on both dedicated cardiac CTs and non-gated chest CTs (9, 11, 36). This capability, often demonstrated in studies with large sample sizes (>1,000 patients), supports the use of AI to accurately identify at-risk individuals in broader populations. Second, AI facilitates advanced risk stratification, moving beyond simple quantification. In this role, AI models integrate the CAC score with other clinical and imaging biomarkers to create prognostic models that can outperform traditional risk scores (10, 37–40). A key future direction for this approach is in refining risk assessment for the “power of zero” cohort. While a CAC score of zero is a powerful marker of low short-term risk, it does not guarantee long-term safety, especially in certain populations (39). AI is uniquely positioned to address this challenge by building models that combine clinical risk factors (such as age, sex, and hypertension) (40) with subtle, subclinical imaging features to identify which patients with a zero score are most likely to experience disease progression. This represents a significant shift from simple detection to proactive, personalized risk management.

We performed a meta-analysis of 9 studies that used AI for detecting ≥50% stenosis, of which most (*n* = 7) were conducted in China and 2 were conducted in the USA. The risk of bias assessment indicated that studies from both regions frequently had an unclear risk of bias in the patient selection domain, often due to insufficient reporting of whether consecutive or random sampling was used. However, studies from the USA tended to provide more detailed methodological descriptions, which facilitated a clearer assessment of bias in some domains. Conversely, several studies from Asia, while often featuring larger sample sizes and advanced AI algorithms, sometimes lacked detailed reporting on patient recruitment and blinding procedures. These differences may reflect variations in research reporting standards, regulatory environments, and access to multicenter data. Overall, our findings highlight the need for greater methodological transparency and harmonization of reporting standards across regions to improve the comparability and generalizability of AI research in cardiovascular imaging.

The meta-analysis of studies that used AI for detecting ≥50% stenosis demonstrated good diagnostic performance of AI applications using both patient- and vessel-level data (combined diagnostic odds ratios of 32.5 and 31.2, and SROC AUCs of 0.83 and 0.92, respectively). However, there was considerable heterogeneity between studies included in the meta-analysis, with I^2^ values for specificity, sensitivity, and diagnostic odds ratio exceeding 60% for patient-level data and 90% for vessel-level data. In terms of the quality of the studies included in the meta-analysis, QUASDAS-2 showed that the risk of bias was generally low for the index test, reference standard, and flow and timing domains, while the risk for the patient selection domain was unclear. The concern regarding applicability of the studies was also low on QUADAS-2, as was the probability of publication bias assessed using Deek's funnel plots (*p* ≤ 0.5).The high degree of heterogeneity (*I*^2^ > 60%) between studies included in the meta-analysis is a critical finding and likely reflects the significant variability in the methodologies and technologies used across the included studies. Several factors may contribute to this. First, the AI algorithms were not uniform, including both in-house academic algorithms and commercially available, sometimes US Food and Drug Administration-cleared, software from vendors such as Shukun Tech and Cleerly (see Table 2). While the presence of commercial tools signals the maturation of AI in cardiovascular imaging, many of these systems function as “black boxes” with proprietary algorithms and training datasets. This hybrid ecosystem highlights a key challenge that direct, head-to-head comparisons of their underlying technology are difficult. Second, there was geographic and demographic variability, with most studies conducted in either China (*n* = 7) or the USA (*n* = 2). Differences in patient populations, disease prevalence, and scanning protocols between these regions could contribute to performance variations. Third, the meta-analysis included mostly single-center and some multicenter retrospective studies, with differing approaches to patient selection. Finally, our quality assessment using QUADAS-2 revealed an unclear risk of bias in the patient selection domain for six of the nine studies (Figure 6A; Supplementary Table 2), suggesting that the lack of clarity on patient selection methods raises the possibility of spectrum bias, where the study population may not be representative of the patients who would typically undergo the test in clinical practice. Therefore, while the pooled results are promising, the high heterogeneity underscores that the diagnostic accuracy of AI is not universal but is highly dependent on the specific algorithm, the clinical setting, and the patient population. While subgroup analysis or meta-regression would typically be used to investigate the sources of this heterogeneity, the small number of included studies (*n* = 9) provides insufficient statistical power for such analyses to be meaningful. Therefore, we can only qualitatively explore the likely drivers based on the characteristics of the included studies (Table 2). There is a need for prospective, multicenter validation studies across diverse populations to confirm the results of the meta-analysis.

The high pooled sensitivity (0.94 at the patient-level) and correspondingly strong negative predictive value (negative likelihood ratio of 0.09) indicate that current AI tools are well-suited for implementation as a triage or rule-out test. In this capacity, AI could rapidly screen CCTA studies to identify patients with a very low likelihood of significant stenosis, allowing human readers to prioritize more complex cases and improve overall workflow efficiency. However, the moderate pooled specificity (0.69 at the patient-level) results in a notable rate of false positives, making these tools less appropriate for standalone diagnosis to confirm disease. Their immediate value is therefore likely as a second reader or decision-support system, where they can assist clinicians by flagging potential lesions for review, thereby reducing inter-observer variability and potentially shortening interpretation times. Although AI has the potential to impact cost-effectiveness by improving efficiency (41, 42), further prospective studies are needed to validate the real-world clinical utility and economic benefits of integrating these tools into routine practice.

Plaque imaging studies were not analyzed with a meta-analysis in our study due to variability between included studies in terms of how AI was used to quantify plaques. This highlights the need for additional studies to assess the diagnostic performance of AI in plaque imaging.

Other recent systematic reviews have also shown the potential for ML and DL techniques to improve the diagnostic and prognostic capabilities of non-invasive cardiovascular imaging, such as prediction of fractional flow reserve (FFR) from CCTA, assessment of coronary artery stenosis, quantification of CAC, and plaque characterization (43, 44). Alskaf et al. conducted a meta-analysis of 8 CT-FFR studies and showed good diagnostic performance of DL applications in the assessment of FFR using CCTA (diagnostic odds ratio 12.5), supporting the increasing use of this diagnostic technique in clinical practice (43).

While AI shows immense potential to enhance clinical practice (44–46), it is crucial to recognize that performance in controlled research settings often does not fully translate to the complexities of real-world clinical workflows. A prime example is the implementation of CT-FFR, where an observational study in England found that its real-world diagnostic accuracy was lower than reported in initial pivotal trials, leading to potentially higher costs than conventional imaging (47). Our own findings reflect this challenge, as several factors can degrade AI performance in practice. For instance, Liu et al. noted that image quality and the level of coronary calcification can significantly impact the accuracy of AI-powered stenosis detection, with performance declining in the presence of severe calcification or image artifacts (23). Similarly, Xu et al. found that certain plaque characteristics, such as composition and lesion length, can affect the diagnostic performance of AI systems (25). These examples illustrate that an AI algorithm trained on a curated dataset may underperform when faced with the full spectrum of patient variability and image quality seen in routine clinical practice. Therefore, post-market surveillance and the validation of AI tools on diverse, “real-world” data are critical steps before their widespread adoption.

Moreover, despite the rapid growth in the application of AI for the diagnosis of CAD, research gaps remain, pointing to the need for future research. A scoping review of studies analyzing the development of diagnostic models of CAD using AI techniques found significant heterogeneity in study design, lack of external validation in almost 90% of AI diagnostic models, and only up to 11% of studies used other data (such as patient demographics and clinical and laboratory data) in addition to image features for AI model development, which may affect the performance of AI in diagnosing CAD (48).

Looking forward, a highly promising application of AI in this field is its use in opportunistic screening for comorbidities that share risk pathways with CAD. A prime example is the automated assessment of non-alcoholic fatty liver disease (NAFLD). As established by Hsiao et al., severe NAFLD is independently associated with an increased risk of subclinical coronary atherosclerosis, highlighting the close relationship between metabolic and cardiovascular disease. An AI tool could automatically quantify liver fat from the attenuation values on a CCTA scan, providing a simultaneous risk assessment for both conditions (49). Similarly, AI can be trained to quantify emphysema and assess lung nodule characteristics, both of which are common comorbidities in patients with cardiovascular risk factors and are pertinent to their overall health and life expectancy (50). By extracting this multi-organ data from a single imaging study, AI has the potential to transform a standard cardiac scan into a comprehensive health screening tool. This approach provides a more holistic view of the patient's health status and enables the early detection of multiple diseases without any extra cost, radiation, or patient time, representing a significant step toward a more integrated and preventive model of medicine.

### 4.1 Strengths and limitations

This systematic review and meta-analysis can be considered a pioneering contribution to the field. Strengths of our study include thorough searches of databases and methodological rigor, including adherence to PRISMA guidelines. We used evidence mapping to summarize and graphically present the geographical distribution of selected studies and the distribution of CT imaging modalities that used AI applications. We also conducted a meta-analysis of both patient-level and vessel-level data to evaluate the diagnostic performance of AI for detecting ≥50% stenosis.

Although our results show good diagnostic performance of AI application in CAD imaging; we acknowledge the limitations of the present study. A key limitation of this review, which reflects the current state of the field, is the significant underrepresentation of non-CT modalities. The small number of CMR studies identified (*n* = 11) precluded any meta-analysis for CMR in our study and highlights a critical gap in the literature that future research should aim to address. There was also insufficient evidence to undertake meta-analyses of other applications of AI in stenosis (for example, the diagnostic performance of AI in detecting ≥70% stenosis and the impact of AI on diagnostic time), and a meta-analysis could not be performed on plaque studies due to variability between studies. The plaque studies varied not only in their endpoints but also in the AI methodologies (e.g., CNN, radiomics-based ML, RCNN), objectives (plaque classification vs. vulnerability assessment vs. risk prediction), and the reference standards used (e.g., expert reader, traditional CCTA, pathology). Additional studies using standardized AI plaque quantification methods are warranted. Other limitations include exclusion of non-English literature, which may introduce language bias, and the high heterogeneity (exceeding 60%) between studies included in the meta-analysis.

In terms of general limitations, AI models are highly sensitive to the data on which they are trained (51–53). Differences in training datasets, data pre-processing methods, and algorithm types can lead to variability in AI performance across studies, which can impact the generalizability of findings. Additionally, many AI applications in imaging are tested on retrospective data or short-term outcomes. Lack of long-term follow-up data in included studies can limit the ability to assess AI's impact on long-term clinical outcomes in cardiovascular disease.

## 5 Conclusion

Evidence mapping showed that the majority of studies using AI-based non-invasive CAD imaging modalities were CT imaging studies, including CT-FFR, plaque or stenosis, and CAC scoring studies. Most studies were conducted in China and the USA, and the majority had sample sizes of 100–500 patients. AI applications demonstrated good diagnostic performance for detecting ≥50% stenosis in a meta-analysis using both patient-level and vessel-level data from patients with known or suspected CAD. A meta-analysis of plaque imaging studies was not undertaken due to variability in how AI was used to quantify plaques, highlighting the need for additional studies to assess the diagnostic performance of AI in plaque imaging.

## Data Availability

The original contributions presented in the study are included in the article/Supplementary Material, further inquiries can be directed to the corresponding author.
