# Supplementary material for: Application of artificial intelligence in non-invasive cardiovascular imaging for coronary artery disease: a systematic review and meta-analysis
Source: Front Cardiovasc Med. 2025 Dec 12;12:1664183. doi: 10.3389/fcvm.2025.1664183 (PMC12741745; doi:10.3389/fcvm.2025.1664183)
Supplement: Supplementary file 1 [file Datasheet1.pdf]

Online Supplement

SEARCH STRATEGIES

Pubmed search strategy

#1. "Cardiovascular Diseases"[Mesh] OR Cardiovascular*[tw] OR Cardiac*[tw] OR heart*[tw] OR ischaemi*[tw] OR ischemi*[tw] OR arrhythmia*[tw] OR coronary artery[tw] OR Atheroscleros*[tw] OR aortic[tw] OR "CT-FFR"[tw]

#2. "Artificial Intelligence"[Mesh] OR AI[tw] OR Artificial Intelligen*[tw] OR Machine Learning*[tw] OR Deep Learning*[tw] OR convolutional neural network*[tw]

#3. "Diagnostic Imaging"[Mesh] OR "diagnostic imaging" [Subheading] OR Imaging*[tw] OR Radiograph*[tw] OR MRI[tw] OR CMR[tw] OR CCTA[tw] OR "CT-FFR"[tw] OR "coronary computed tomography angiograph*"[tw] OR "cardiovascular magnetic resonance imaging"[tw]

#4. #1 AND #2 AND #3 AND ("2018/01/01"[Date - Publication] : "3000"[Date - Publication]) 5725

#5. "Meta-Analysis"[pt] OR "Meta-Analysis as Topic"[Mesh] OR "Systematic Review" [pt] OR "Systematic Reviews as Topic"[Mesh] OR "systematic"[Filter] OR "Systematic Review*"[tiab] OR "Meta-Analysis"[tiab] OR Metaanalys*[tiab]

#6. ("Clinical Trial" [pt] OR "Observational Study" [pt] OR "Clinical Trials as Topic"[Mesh] OR "Observational Studies as Topic"[Mesh] OR "Random Allocation"[MeSH] OR "Double-Blind Method"[MeSH] OR "single-blind method"[MeSH] OR "Control Groups"[MeSH] OR "cross-over studies"[MeSH] OR "Cohort Studies"[Mesh] OR "Case-Control Studies"[Mesh] OR "Cross-Sectional Studies"[Mesh] OR "Pragmatic Clinical Trials as Topic"[Mesh] OR "Real World"[tw]

OR random*[tiab] OR placebo[tiab] OR trial[tiab] OR groups[tiab] OR crossover[tiab] OR cross-over[tiab] OR Observational Stud*[tiab] OR Cohort[tiab] OR Follow-Up[tiab] OR Longitudinal*[tiab] OR Prospectiv*[tiab] OR Retrospectiv*[tiab] OR Case-Control[tiab] OR Cross-Sectional[tiab] OR "controlled trial"[tiab] OR "controlled study"[tiab] OR "single arm"[tiab] OR Feasibility study[tiab] OR "proof of concept"[tiab] OR Clinical validation*[tiab] OR Preliminary validation*[tiab]) NOT ("Animals"[Mesh] NOT ("Humans"[Mesh] AND "Animals"[Mesh]))

#7. #4 AND (#5 OR #6) 2709

#8. coronary artery calcium*[tw] OR coronary artery calcification*[tw]

#9. "Atherosclerosis"[Mesh] OR Atheroscleros*[tw] OR Atherogenes*[tw] OR "Coronary Stenosis"[Mesh] OR Coronary Stenos*[tw] OR Coronary Artery Stenos*[tw] OR coronary restenos*[tw]

#10. "Fractional Flow Reserve, Myocardial"[Mesh] OR "CT-FFR"[tw] OR fractional flow reserve*[tw]

#11. (Cardiovascular*[tw] OR Cardiac*[tw] OR heart*[tw]) AND ("Magnetic Resonance Imaging"[Mesh] OR MRI[tw] OR NMR Imaging*[tw] OR MR Tomograph*[tw] OR NMR Tomograph*[tw] OR Magnetic Resonance Image*[tw])

#12. "Aortic Diseases"[Mesh] OR aortic disease*[tw] OR aortic[tw] OR Aortiti*[tw]

#13.#7 AND (#8 OR #9 OR #10 OR #11 OR #12) 958

EMBASE search strategy

#1. 'cardiovascular disease'/exp OR (Cardiovascular* OR Cardiac* OR heart* OR ischaemi* OR ischemi* OR arrhythmia* OR "coronary artery" OR Atheroscleros* OR aortic OR "CT-FFR"):ab,ti,kw

#2. 'artificial intelligence'/exp OR (AI OR "Artificial Intelligen*" OR "Machine Learning*" OR "Deep Learning*" OR "convolutional neural network*"):ab,ti,kw

#3. 'diagnostic imaging'/exp OR (Imaging* OR Radiograph* OR MRI OR CMR OR CCTA OR "CT-FFR" OR "coronary computed tomography angiograph*" OR "cardiovascular magnetic resonance imaging"):ab,ti,kw

#4. #1 AND #2 AND #3 AND [2018-2023]/py 6407

#5. 'meta analysis'/exp OR 'meta analysis (topic)'/exp OR 'systematic review'/exp OR 'systematic review (topic)'/exp OR (Systemat* NEAR/3 Review* OR 'Meta Analysis*' OR Metaanalys*):ab,ti,kw

#6. ('clinical trial'/exp OR 'Clinical Trial (Topic)'/exp OR 'double blind procedure'/de OR 'control group'/de OR 'crossover procedure'/de OR 'single blind procedure'/de OR 'triple blind procedure'/de OR 'placebo'/de OR 'randomization'/exp OR 'observational study'/exp OR 'cohort analysis'/exp OR 'cross-sectional study'/exp OR 'case control study'/exp OR 'pragmatic trial'/exp OR (random* OR trial OR groups OR placebo* OR crossover OR "cross-over" OR "observational stud*" OR Cohort OR "Follow-Up" OR Longitudinal* OR Prospectiv* OR Retrospectiv* OR "Case-Control" OR "Cross-Sectional" OR "case-series" OR "single arm" OR "Real World" OR "Feasibility study" OR "proof of concept" OR "Clinical validation*" OR "Preliminary validation*"):ab,ti,kw) NOT (('nonhuman'/exp OR 'animal'/exp) NOT 'human'/exp)

#7. #4 AND (#5 OR #6) 3317

#8. 'coronary artery calcification'/exp OR 'atherosclerosis'/exp OR 'coronary stenosis'/exp OR (Atheroscleros* OR Atherogenes* OR (Coronary NEAR/3 (calcium* OR calcification* OR Stenos* OR restenos*))):ab,ti,kw

#9. 'fractional flow reserve'/exp OR ("CT-FFR" OR "fractional flow reserv*"):ab,ti,kw

#10. ((Cardiovascular* OR Cardiac* OR heart*):ab,ti,kw) AND ('nuclear magnetic resonance imaging'/exp OR (MRI OR "NMR Imaging*" OR "MR Tomograph*" OR "NMR Tomograph*" OR "Magnetic Resonance Image*"):ab,ti,kw)

#11. 'aortic disease'/exp OR ((aortic NEAR/3 disease*) OR aortic OR Aortiti*):ab,ti,kw

#12.#7 AND (#8 OR #9 OR #10 OR #11) 1278

Web of Science search strategy

#1. TS=(Cardiovascular* OR Cardiac* OR heart* OR ischaemi* OR ischemi* OR arrhythmia* OR "coronary artery" OR Atheroscleros* OR aortic OR "CT-FFR")

#2. TS=(AI OR "Artificial Intelligen*" OR "Machine Learning*" OR "Deep Learning*" OR "convolutional neural network*")

#3. TS=(Imaging* OR Radiograph* OR MRI OR CMR OR CCTA OR "CT-FFR" OR "coronary computed tomography angiograph*" OR "cardiovascular magnetic resonance imaging")

#4. #1 AND #2 AND #3 AND [2018-2023]/py 3817

#5. TS=("Meta-Analysis" OR "Meta Analysis" OR "Systematic Review" OR "Systematic Review*" OR "Meta-Analysis" OR "Meta-Analy*" OR "Metaanaly*" OR ("systematic*" NEAR/4 "Review*"))

#6. TS=(((Random* OR Clinical OR controlled) NEAR/3 Trial) OR "Random Allocation" OR Randomization OR "Double Blind" OR "single blind" OR "Control Group" OR "Control Groups" OR random* OR trial OR groups OR placebo* OR crossover OR "cross-over" OR "observational stud*" OR Cohort OR "Follow-Up" OR Longitudinal* OR Prospectiv* OR Retrospectiv* OR "Case-Control" OR "Cross-Sectional" OR "case-series" OR "single arm" OR "Real World" OR "Feasibility study" OR "proof of concept" OR "Clinical validation*" OR "Preliminary validation*")

#7. #4 AND (#5 OR #6) 1559

#8. TS=(Atheroscleros* OR Atherogenes* OR (Coronary NEAR/3 (calcium* OR calcification* OR Stenos* OR restenos*)))

#9. TS=("CT-FFR" OR "fractional flow reserv*")

#10. (TS=(Cardiovascular* OR Cardiac* OR heart*)) AND (TS=(MRI OR "NMR Imaging*" OR "MR Tomograph*" OR "NMR Tomograph*" OR "Magnetic Resonance Image*"))

#11. TS=((aortic NEAR/3 disease*) OR aortic OR Aortiti*)

#12.#7 AND (#8 OR #9 OR #10 OR #11) 676

Engineering Index search strategy

#1. (Cardiovascular* OR Cardiac* OR heart* OR ischaemi* OR ischemi* OR arrhythmia* OR "coronary artery" OR Atheroscleros* OR aortic OR "CT-FFR")

#2. (AI OR "Artificial Intelligen*" OR "Machine Learning*" OR "Deep Learning*" OR "convolutional neural network*")

#3. (Imaging* OR Radiograph* OR MRI OR CMR OR CCTA OR "CT-FFR" OR "coronary computed tomography angiograph*" OR "cardiovascular magnetic resonance imaging")

#4. ("Meta-Analysis" OR "Meta Analysis" OR "Systematic Review" OR "Systematic Review*" OR "Meta-Analysis" OR "Meta-Analy*" OR "Metaanaly*" OR ("systematic*" NEAR/4 "Review*") OR (Random* NEAR/3 Trial) OR (Clinical NEAR/3 Trial) OR (controlled NEAR/3 Trial) OR "Random Allocation" OR Randomization OR "Double Blind" OR "single blind" OR "Control Group" OR "Control Groups" OR random* OR trial OR groups OR placebo* OR crossover OR "cross-over" OR "observational stud*" OR Cohort OR "Follow-Up" OR Longitudinal* OR Prospectiv* OR Retrospectiv* OR "Case-Control" OR "Cross-Sectional" OR "case-series" OR "single arm" OR "Real World" OR "Feasibility study" OR "proof of concept" OR "Clinical validation*" OR "Preliminary validation*")

#5. (Atheroscleros* OR Atherogenes* OR (Coronary NEAR/3 calcium*) OR (Coronary NEAR/3 calcification*) OR (Coronary NEAR/3 Stenos*) OR (Coronary NEAR/3 restenos*) OR "CT-FFR" OR "fractional flow reserv*" OR ((Cardiovascular* OR Cardiac* OR heart*) AND (MRI OR "NMR Imaging*" OR "MR Tomograph*" OR "NMR Tomograph*" OR "Magnetic Resonance Image*")) OR (aortic NEAR/3 disease*) OR aortic OR Aortiti*)

#6. #1 AND #2 AND #3 AND #4 AND #5 AND (2018-2023) 304

Cochrane Library search strategy

#1 MeSH descriptor: [Cardiovascular Diseases] explode all trees 151802

#2 (Cardiovascular* OR Cardiac* OR heart* OR ischaemi* OR ischemi* OR arrhythmia* OR "coronary artery" OR Atheroscleros* OR aortic OR "CT-FFR"):ti,ab,kw 295449

#3 #1 or #2 348520

#4 MeSH descriptor: [Artificial Intelligence] explode all trees 2929

#5 (AI OR "Artificial Intelligen*" OR "Machine Learning*" OR "Deep Learning*" OR "convolutional neural network*"):ti,ab,kw 8582

#6 #4 or #5 10356

#7 MeSH descriptor: [Diagnostic Imaging] explode all trees 63035

#8 MeSH descriptor: [] explode all trees and with qualifier(s): [diagnostic imaging - DG] 36349

#9 (Imaging* OR Radiograph* OR MRI OR CMR OR CCTA OR "CT-FFR" OR "coronary computed tomography angiograph*" OR "cardiovascular magnetic resonance imaging"):ti,ab,kw 118262

#10 #7 or #8 or #9 137536

#11 #3 and #6 and #10 438

#12 MeSH descriptor: [Atherosclerosis] explode all trees 4455

#13 MeSH descriptor: [Coronary Stenosis] explode all trees 1872

#14 MeSH descriptor: [Aortic Diseases] explode all trees 1398

#15 (Atheroscleros* OR Atherogenes* OR (Coronary NEAR/3 (calcium* OR calcification* OR Stenos* OR restenos*))):ti,ab,kw 15375

#16 ("CT-FFR" OR "fractional flow reserv*" OR (aortic NEAR/3 disease*) OR aortic OR Aortiti*):ti,ab,kw 11553

#17 (Cardiovascular* OR Cardiac* OR heart*):ti,ab,kw 252813

#18 MeSH descriptor: [Magnetic Resonance Imaging] explode all trees 10939

#19 (MRI OR "NMR Imaging*" OR "MR Tomograph*" OR "NMR Tomograph*" OR "Magnetic Resonance Image*"):ti,ab,kw 32390

#20 #18 OR #19 37367

#21 #17 AND #20 4581

#22 #12 OR #13 OR #14 OR #15 OR #16 OR #21 32235

#23 #11 AND #22 173，均为 trials

Fig S1 Diagnostic performance of AI to detect ≥50% stenosis using patient-level data. Forest plots of (**a**) likelihood ratio and (**b**) diagnostic odds ratio

a

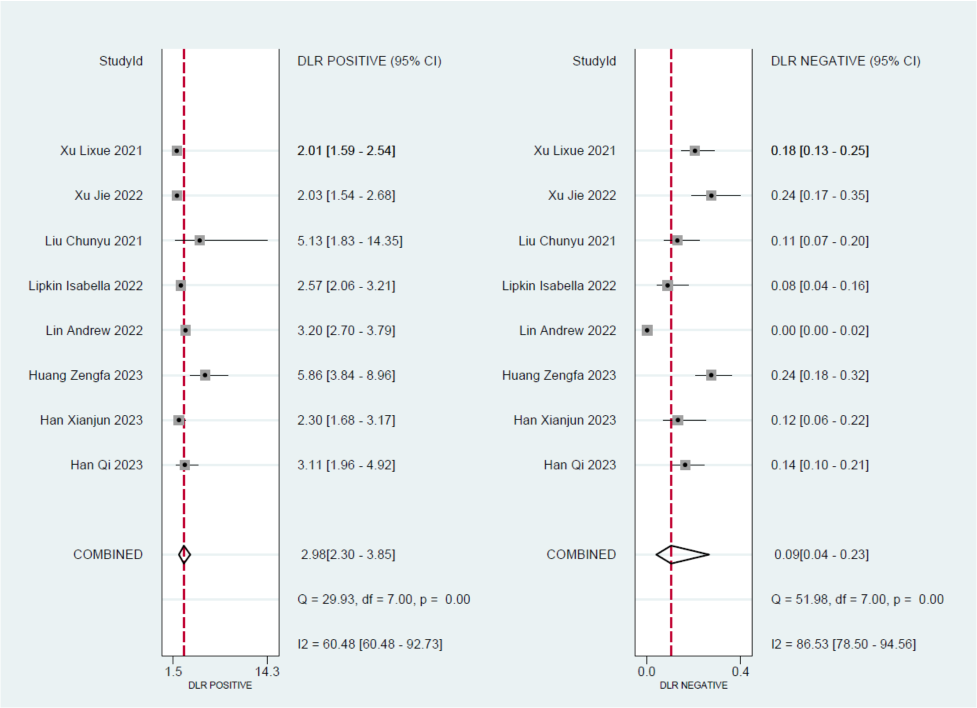

b

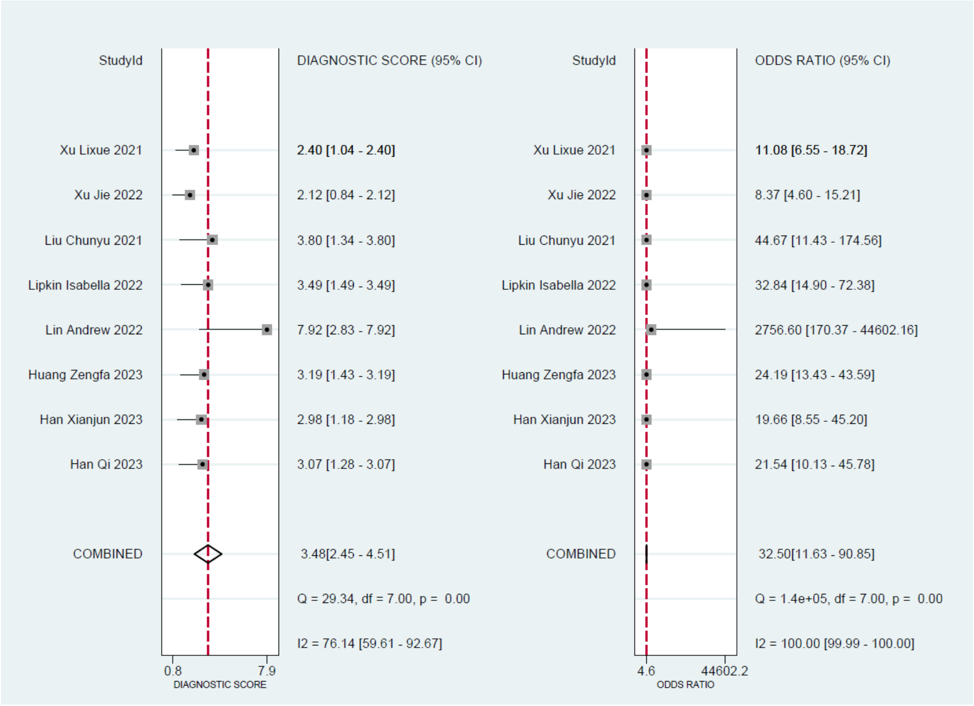

Fig S2 Scatter plots showing the summary points of positive and negative likelihood ratios from (**a**) patient-level data and (**b**) vessel-level data

a

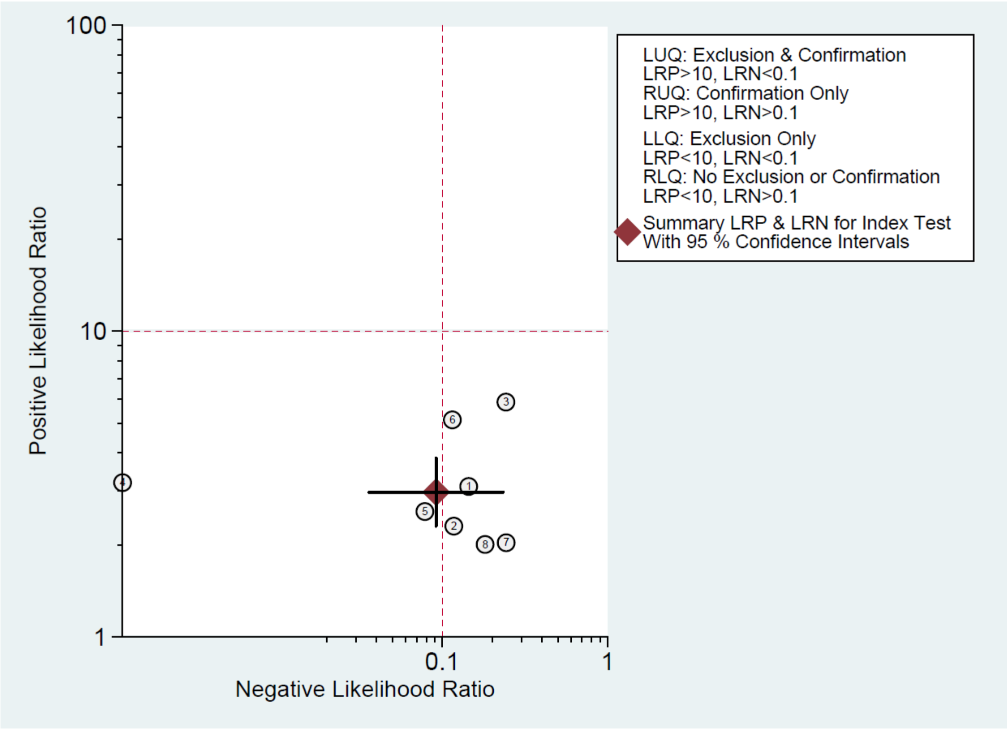

b

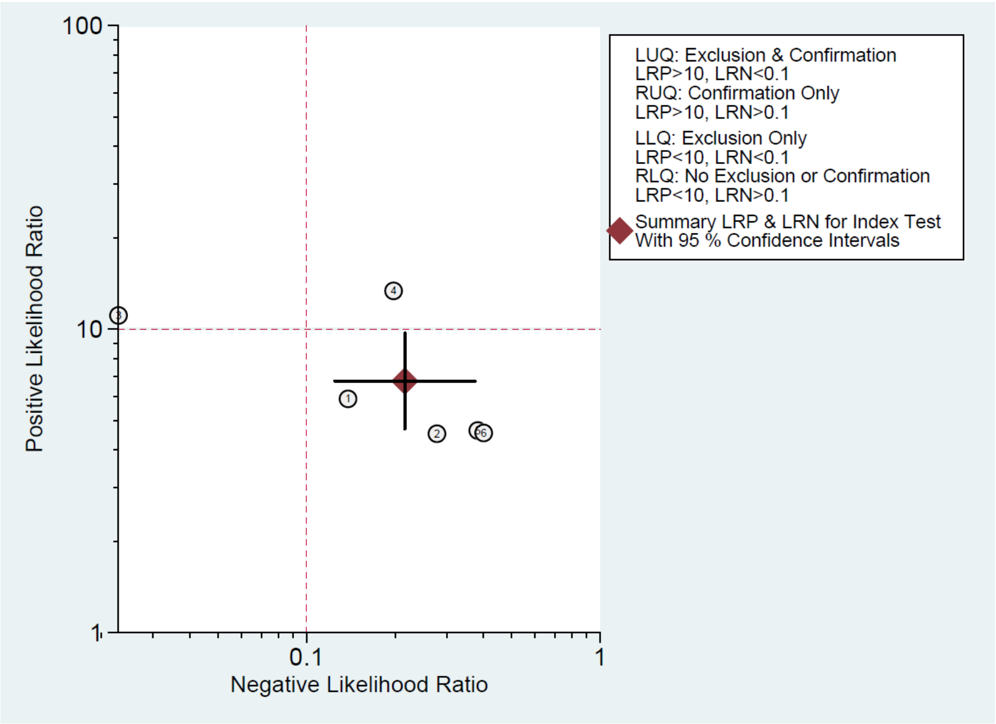

Fig. S3 Fagan's nomogram for computation of pre- and post-test probability using (**a**) patient-level and (**b**) vessel-level data

a

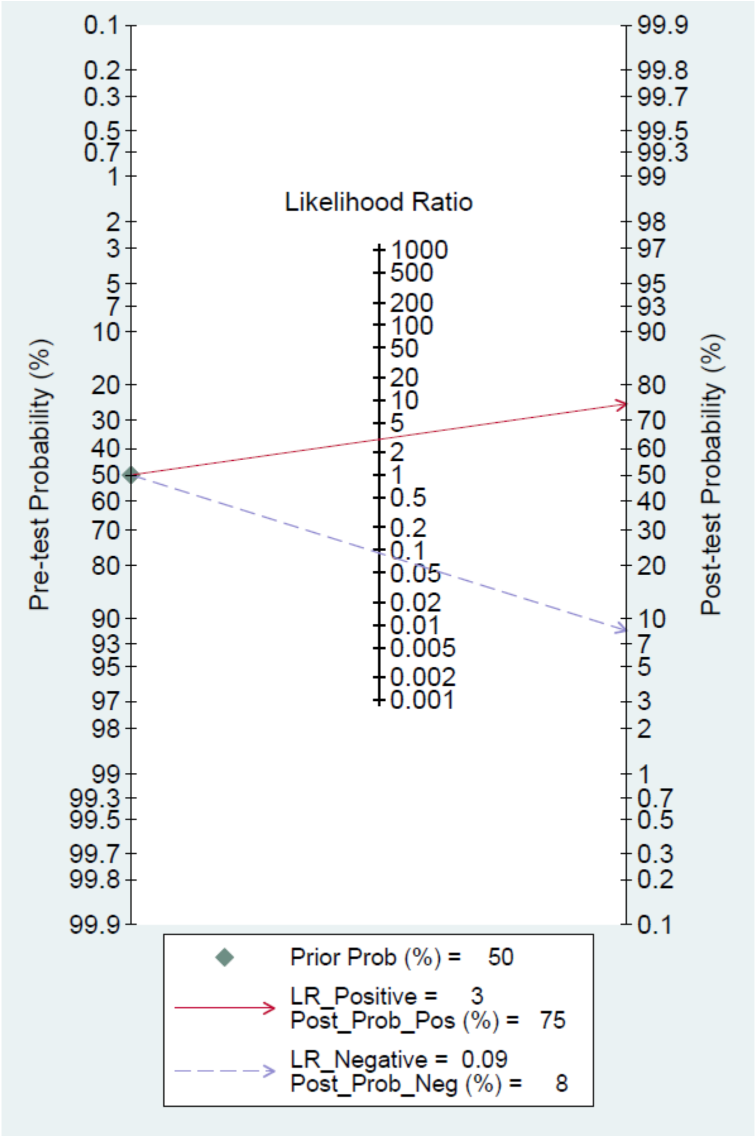

b

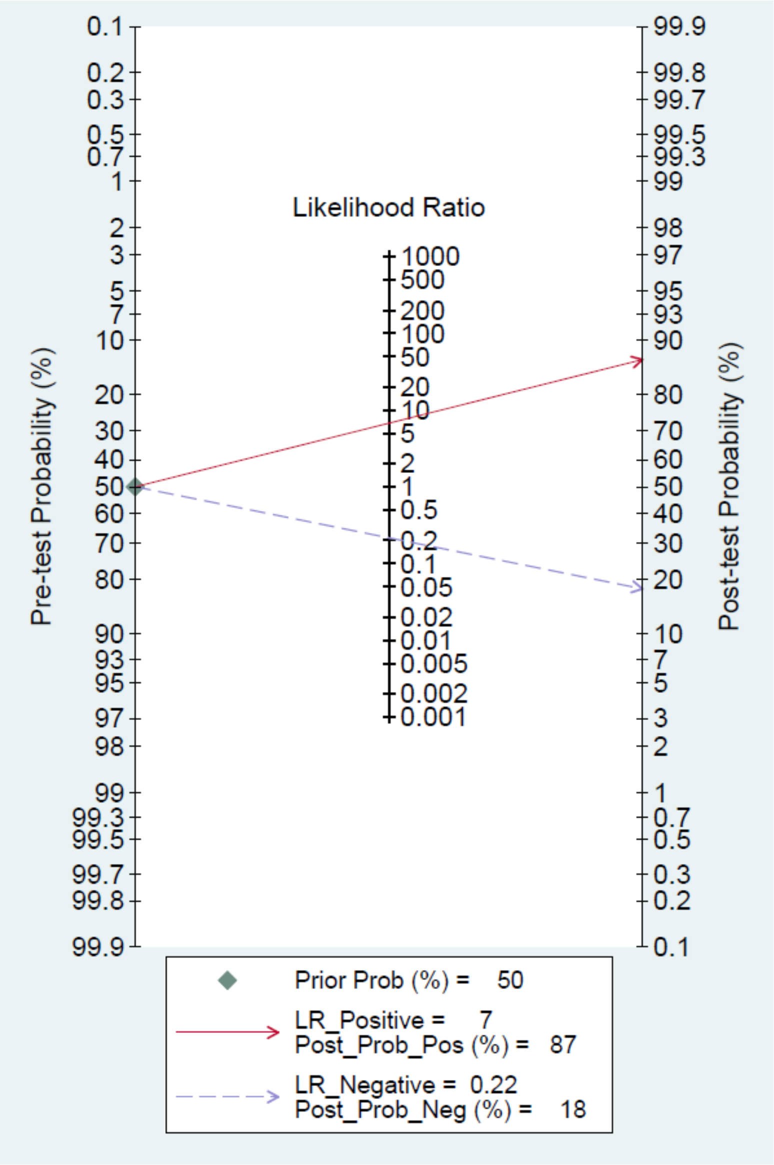

Pre-test Probability (%)
Likelihood Ratio
Post-test Probability (%)
0.1
0.2
0.3
0.5
0.7
1
2
3
5
7
10
20
30
40
50
60
70
80
90
93
95
97
98
99
99.3
99.5
99.7
99.8
99.9
1000
500
200
100
50
20
10
5
2
1
0.5
0.2
0.1
0.05
0.02
0.01
0.005
0.002
0.001
Prior Prob (%) = 50
LR_Positive = 7
Post_Prob_Pos (%) = 87
LR_Negative = 0.22
Post_Prob_Neg (%) = 18

Fig S4 Diagnostic performance of AI to detect ≥50% stenosis using vessel-level data. Forest plots of (**a**) likelihood ratio and (**b**) diagnostic odds ratio

a

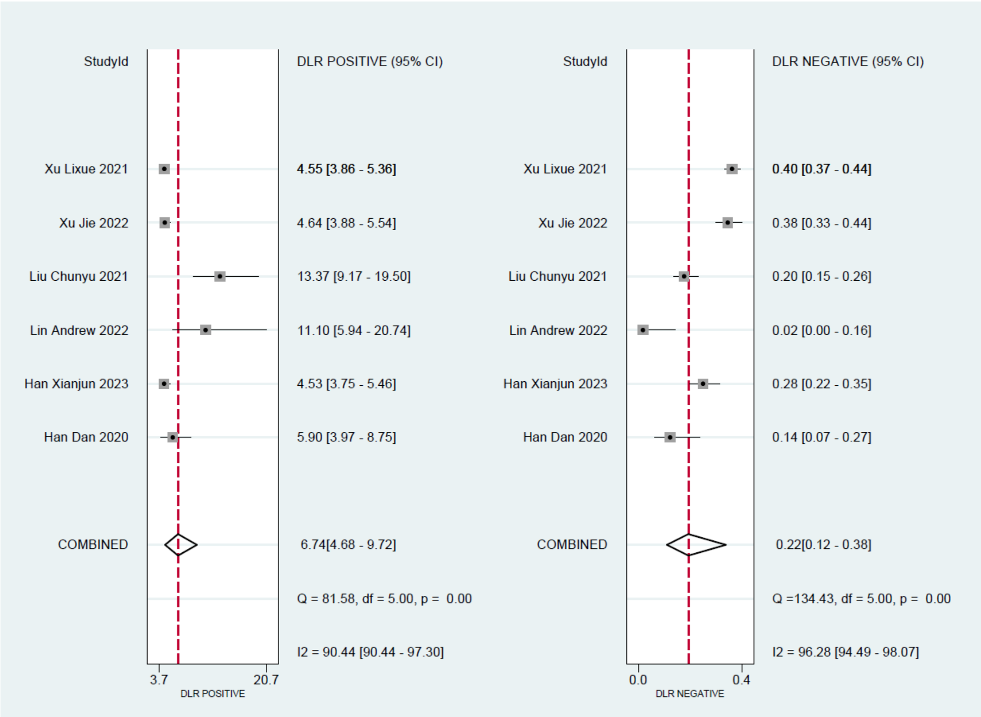

b

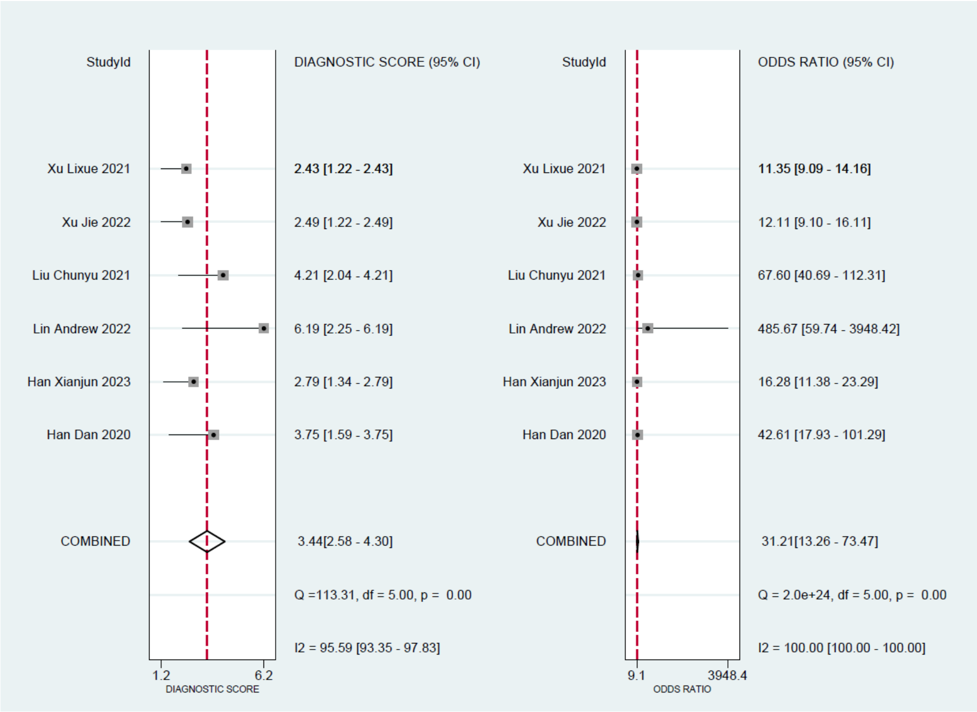

Table S1. Characteristics of evidence mapping studies

ID	Year	Study	Study design	Sample Size	Indications-final	Modality	Location	Type of study	Type of AI-specific
**1**	2021	Liu et al. (1)	Retrospective	296	CT-FFR	CT	China	Diagnosis	DL
**2**	2018	Sakellarios et al. (2)	Prospective/ Retrospective	250	CT-FFR	CT	Singapore	Diagnosis and Predictive factor	ML
**3**	2022	Li et al. (3)	Retrospective	73	CT-FFR	CT	China	Diagnosis and Predictive factor	DL
**4**	2022	Gohmann et al. (4)	Retrospective	460	CT-FFR	CT	Germany	Diagnosis	ML
**5**	2022	Ghorashi et al. (5)	Retrospective	500	CT-FFR	CT	Iran	Predictive model	ML
**6**	2018	Tesche et al. (6)	Retrospective	85	CT-FFR	CT	Germany	Diagnosis	ML
**7**	2019	von Knebel Doeberitz et al. (7)	Retrospective	84	CT-FFR	CT	USA	Diagnosis and Predictive factor	DL/ ML
**8**	2018	Yu et al. (8)	Retrospective	129	CT-FFR	CT	China	Diagnosis and Predictive model	ML
**9**	2020	Zreik et al. (9)	Retrospective	187	CT-FFR	CT	Netherlands	Diagnosis	DL
**10**	2019	van Hamersvelt et al. (10)	Retrospective	126	CT-FFR	CT	Netherlands	Diagnosis	DL
**11**	2018	Zreik et al. (11)	Retrospective	166	CT-FFR	CT	Netherlands	Diagnosis	DL/ML
**12**	2019	Li et al. (12)	Prospective	86	CT-FFR	MPI and CT	China	Diagnosis	ML
**13**	2020	Kumamaru et al. (13)	Retrospective	1052	CT-FFR	CT	Japan	Diagnosis	DL
**14**	2018	Coenen et al. (14)	Retrospective	351	CT-FFR	CT	USA	Diagnosis	DL/ML
**15**	2022	Zhang et al. (15)	Retrospective	36	CT-FFR	CT	China	Diagnosis	DL
**16**	2019	Tang et al. (16)	Retrospective	136	CT-FFR	CT	China	Diagnosis	ML
**17**	2018	Hu et al. (17)	Retrospective	105	CT-FFR	CT	China	Diagnosis	ML
**18**	2020	Yu et al. (18)	Retrospective	167	CT-FFR	CT	China	Diagnosis and Predictive model	ML
**19**	2023	Yang et al. (19)	Prospective/ Retrospective	146	CT-FFR	CT	China	Diagnosis	ML
**20**	2019	De Geer et al. (20)	Retrospective	351	CT-FFR	CT	Sweden	Diagnosis	ML

**21**	2020	Kawasaki et al. (21)	Retrospective	47	CT-FFR	CT	Japan	Diagnosis and Predictive model	ML
**22**	2022	Xue (22)	Retrospective	40	CT-FFR	CT	China	Diagnosis	DL
**23**	2023	Pan et al. (23)	Retrospective	229	CT-FFR	CT	China	Diagnosis	DL
**24**	2019	von Knebel Doeberitz et al. (24)	Retrospective	82	CT-FFR	CT	Germany	Predictive model	ML
**25**	2020	Qiao et al. (25)	Retrospective	1121	CT-FFR	CT	China	Predictive factor	ML
**26**	2020	Nous et al. (26)	Retrospective	372	CT-FFR	CT	Netherlands	Diagnosis	ML
**27**	2021	Renker et al. (27)	Retrospective	330	CT-FFR	CT	USA	Diagnosis	ML
**28**	2020	Gao et al. (28)	Retrospective	180	CT-FFR	CT	China	Diagnosis	DL/ML
**29**	2022	Feng et al. (29)	Retrospective	350	CT-FFR	CT	China	Diagnosis and Predictive model	ML
**30**	2022	Qiao (30)	Prospective	1133	CT-FFR	CT	China	Predictive factor	ML
**31**	2023	Yang et al. (31)	Retrospective	1216	CT-FFR	CT	China	Predictive factor	DL/ML (Keya)
**32**	2019	Kurata et al. (32)	Retrospective	74	CT-FFR	CT	Japan	Diagnosis	ML
**33**	2022	Lee et al. (33)	Retrospective	NR	CT-FFR	CT	S. Korea	Diagnosis and Predictive model	DL/ML (Keya)
**34**	2020	Qiao et al. (34)	Retrospective	NA	CT-FFR	CT	China	Predictive factor	ML
**35**	2022	Ahmed et al. (35)	Retrospective	471	CT-FFR	CT and SPECT MPI	USA	Predictive factor	ML
**36**	2019	van Assen et al. (36)	Retrospective	81	CT-FFR	CT and CTP	USA	Predictive factor	AI
**37**	2022	Brandt et al. (37)	Retrospective	117	CT-FFR	CT	Germany	Predictive factor	ML
**38**	2022	Al Rifai et al. (38)	Retrospective	471	CT-FFR	CT and SPECT	USA	Diagnosis and Predictive factor	ML
**39**	2022	Lossnitzer et al. (39)	Retrospective	141	CT-FFR	CT	Germany	Diagnosis	ML
**40**	2022	Tang et al. (40)	Retrospective	NR	CT-FFR	CT	USA	Diagnosis and Predictive model	DL/ML (Keya)
**41**	2019	Yu et al. (41)	Retrospective	180	CT-FFR	CT	China	Diagnosis and Predictive model	ML
**42**	2021	Di Jiang et al. (42)	Retrospective	442	CT-FFR	CT	China	Diagnosis	ML

**43**	2022	Xu at al. (43)	Retrospective	33	CT-FFR	CT	China	Diagnosis	DL/ML
**44**	2020	Li et al. (44)	Retrospective	107	CT-FFR	CT	China	Diagnosis	ML
**45**	2020	Xu et al. (45)	Retrospective	437	CT-FFR	CT	China	Diagnosis	ML
**46**	2020	Martin et al. (46)	Retrospective	159	CT-FFR	CT	USA	Diagnosis and Predictive factor	ML
**47**	2019	Yang et al. (47)	Retrospective	NR	CMR	CMR	China	Diagnosis	DL
**48**	2022	Morales et al. (48)	Prospective	503	CMR	CMR	USA	Diagnosis	CNN
**49**	2019	Zhang et al. (49)	Retrospective	299	CMR	CMR	China	Diagnosis	DL
**50**	2023	Masutani et al. (50)	Retrospective	223	CMR	CMR	USA	Diagnosis	DL
**51**	2020	Scannell at al. (51)	Retrospective	175	CMR	CMR/MPI	UK	Diagnosis	DL
**52**	2022	Morales et al. (52)	Prospective	119	CMR	CMR	USA	Diagnosis	DL
**53**	2020	Böttcher et al. (53)	Retrospective	50	CMR	CMR	Germany	Diagnosis	DL
**54**	2023	Zaidi et al. (54)	Prospective	397	CMR	CMR	UK	Predictive model	ML
**55**	2022	Pezel et al. (55)	Retrospective	31752	CMR	CMR	France	Predictive model	ML
**56**	2023	Pujadas et al. (56)	Retrospective	NR	CMR	CMR	UK	Predictive model	ML
**57**	2022	Yan et al. (57)	Retrospective	1354	CMR	CMR	China	Diagnosis	CNN
**58**	2022	Klug et al. (58)	Prospective	59	Calcium scoring (CAC)	Chest CT	Israel	Diagnosis	CNN
**59**	2021	Zeleznik et al. (59)	Retrospective	20084	Calcium scoring (CAC)	Cardiac CT	USA	Predictive factor	Complex-valued convolutional network
**60**	2023	Suh et al. (60)	Retrospective	452	Calcium scoring (CAC)	Cardiac CT and Chest CT	S. Korea	Diagnosis	AI
**61**	2020	Fischer et al. (61)	Retrospective	194	Calcium scoring (CAC)	CT	USA	Diagnosis	RNN-LSTM
**62**	2023	Peng et al. (62)	Retrospective	5678	Calcium scoring (CAC)	Chest CT	USA	Predictive factor	DL
**63**	2021	Chamberlin et al. (63)	Retrospective	117	Calcium scoring (CAC)	Chest CT	USA	Diagnosis and Predictive factor	CNN
**64**	2022	Yu et al. (64)	Retrospective	405	Calcium scoring (CAC)	Cardiac CT and Chest CT	China	Diagnosis	AI
**65**	2021	van Assen et al. (65)	Retrospective	263	Calcium scoring (CAC)	Cardiac CT and Chest CT	USA	Diagnosis	DL CNN

**66**	2022	Mu et al. (66)	Retrospective	365	Calcium scoring (CAC)	CT	China	Diagnosis	DL
**67**	2020	Wang et al. (67)	Retrospective	530	Calcium scoring (CAC)	Cardiac CT	China	Diagnosis	DL
**68**	2020	van Velzen et al. (68)	Retrospective	7240	Calcium scoring (CAC)	Cardiac CT and Chest CT	Netherlands	Diagnosis	DL
**69**	2022	Winkel et al. (69)	Retrospective	1171	Calcium scoring (CAC)	Cardiac CT	USA	Diagnosis	DL
**70**	2023	Lee et al. (70)	Retrospective	315	Calcium scoring (CAC)	CT	S. Korea	Diagnosis	DL
**71**	2020	Sandstedt et al. (71)	Retrospective	342	Calcium scoring (CAC)	Cardiac CT	Sweden	Diagnosis	AI
**72**	2021	Lee et al. (72)	Retrospective	2985	Calcium scoring (CAC)	Cardiac CT and ct	S. Korea	Diagnosis	DL
**73**	2021	Zhang et al. (73)	Retrospective	232	Calcium scoring (CAC)	Cardiac CT	China	Diagnosis	DL
**74**	2023	Inoue et al. (74)	Retrospective	3328	Calcium scoring (CAC)	CT	Japan	Predictive factor	ML
**75**	2022	Barda et al. (75)	Retrospective	470	Calcium scoring (CAC)	Chest CT	USA	Predictive model	ML
**76**	2021	Nakanishi (76)	Retrospective	66,636	Calcium scoring (CAC)	Cardiac CT	USA	Predictive model	ML
**77**	2021	Tamarappoo et al. (77)	Prospective	1069	Calcium scoring (CAC)	Cardiac CT	USA	Predictive model	ML
**78**	2020	Al'Aref at al. (78)	Retrospective	13054	Calcium scoring (CAC)	CT	USA	Predictive model	ML
**79**	2023	Yu et al. (79)	Retrospective	184	Calcium scoring (CAC)	Cardiac CT	China	Predictive model	ML
**80**	2019	Commandeur et al. (80)	Prospective	2071	Calcium scoring (CAC)	Cardiac CT	USA	Predictive model	ML
**81**	2020	Glowacki et al. (81)	Retrospective	435	Calcium scoring (CAC)	CT	Poland	Diagnosis	ML
**82**	2018	Cano-Espinosa et al. (82)	Retrospective	6983	Calcium scoring (CAC)	Chest CT	Spain	Diagnosis	DL
**83**	2023	Dobrolinska et al. (83)	Retrospective	213	Calcium scoring (CAC)	Chest CT	Netherlands	Diagnosis	DL
**84**	2020	Eslami et al. (84)	Retrospective	624	Calcium scoring (CAC)	Cardiac CT	Germany	Predictive factor	ML
**85**	2022	Huang et al. (85)	Retrospective	NR	Calcium scoring (CAC)	CT	China	Predictive model	ML
**86**	2018	Lessmann et al. (86)	Retrospective	1744	Calcium scoring (CAC)	Chest CT	Netherlands	Diagnosis	CNN
**87**	2018	Zreik et al. (87)	Retrospective	163	Plaques or stenosis	CT	Netherlands	Diagnosis	CNN
**88**	2023	Penso et al. (88)	Retrospective	288	Plaques or stenosis	CT	Italy	Diagnosis	Token-mixer architecture (ConvMixer)
**89**	2022	Sun et al. (89)	Retrospective	50	Plaques or stenosis	CT	Australia	Diagnosis	Generative adversarial network (ESRGAN)

**90**	2022	Han et al. (90)	Retrospective	196	Plaques or stenosis	CT	China	Diagnosis	DL
**91**	2023	Kim et al. (91)	Retrospective	747	Plaques or stenosis	CT	USA	Diagnosis	ML/AI-QCT
**92**	2022	Yunus et al. (92)	Retrospective	202	Plaques or stenosis	CT	India	Diagnosis	ML/Auto-WEKA
**93**	2022	Li et al. (93)	Retrospective	443	Plaques or stenosis	CT	China	Diagnosis	DL
**94**	2022	Jin et al. (94)	Retrospective	505	Plaques or stenosis	CT	China	Diagnosis	CNN
**95**	2023	Huang et al. (95)	Retrospective	346	Plaques or stenosis	CT	China	Diagnosis	CNN
**96**	2023	Huang et al. (96)	Retrospective	1796	Plaques or stenosis	CT	China	Predictive factor	CNN
**97**	2022	Lipkin et al. (97)	Prospective/Retrospective	301	Plaques or stenosis	CT	USA	Diagnosis	ML/ AI-QCT
**98**	2020	Han et al. (98)	Retrospective	150	Plaques or stenosis	CT	China	Diagnosis	CNN
**99**	2021	Liu et al. (99)	Retrospective	165	Plaques or stenosis	CT	China	Diagnosis	DL
**100**	2023	Wu et al. (100)	Prospective	64	Plaques or stenosis	MRA	China	Diagnosis	DL
**101**	2023	Lee et al. (101)	Retrospective	11180	Plaques or stenosis	CT	S. Korea	Predictive model	DL/ML
**102**	2022	Lin et al. (102)	Prospective/Retrospective	921	Plaques or stenosis	CT	USA	Diagnosis and Predictive factor	CNN
**103**	2021	Xu et al. (103)	Retrospective	527	Plaques or stenosis	CT	China	Diagnosis	DL
**104**	2022	Xu et al. (104)	Retrospective	306	Plaques or stenosis	CT	China	Diagnosis	AI
**105**	2023	Mátyás et al. (105)	Retrospective	158	Plaques or stenosis	CT	Romania	Diagnosis	AI
**106**	2023	Fagman et al. (106)	Retrospective	469	Plaques or stenosis	CT	Sweden	Diagnosis	DL
**107**	2022	Li et al. (107)	Retrospective	44	Plaques or stenosis	CT	China	Diagnosis	ML
**108**	2021	Tesche et al. (108)	Retrospective	361	Plaques or stenosis	CT	Germany	Predictive model	ML
**109**	2022	Dou et al. (109)	Retrospective	18	Plaques or stenosis	CT	China	Predictive model	ML
**110**	2022	Jonas et al. (110)	Retrospective	232	Plaques or stenosis	CT	USA	Diagnosis	AI
**111**	2018	van Rosendael et a. (111)	Retrospective	8844	Plaques or stenosis	CT	USA	Predictive model	ML
**112**	2023	Li et al. (112)	Retrospective	240	Plaques or stenosis	CT	China	Predictive model	ML
**113**	2020	Muscogiuri et al. (113)	Retrospective	288	Plaques or stenosis	CT	Italy	Diagnosis	CNN
**114**	2022	Shu et al. (114)	Retrospective	154	Plaques or stenosis	CT	China	Predictive model	ML
**115**	2022	Benz et al. (115)	Prospective	50	Plaques or stenosis	CT	Switzerland	Diagnosis	DL

**116**	2022	Lin et al. (116)	Prospective	60	Plaques or stenosis	CT	USA	Diagnosis and Predictive model	ML
**117**	2023	Jonas et al. (117)	Retrospective	303	Plaques or stenosis	CT	USA	Diagnosis	AI
**118**	2022	Wang et al. (118)	Retrospective	452	Plaques or stenosis	CT	China	Predictive model	ML
**119**	2022	Jonas et al. (119)	Retrospective	303	Plaques or stenosis	CT	USA	Diagnosis	AI/AI-QCT
**120**	2023	Han et al. (120)	Retrospective	200	Plaques or stenosis	CT	China	Diagnosis	AI
**121**	2023	Han et al. (121)	Retrospective	318	Plaques or stenosis	CT	China	Diagnosis	DL
**122**	2021	Li et al. (122)	Retrospective	149	Plaques or stenosis	CT	China	Diagnosis and Predictive model	ML

AI=artificial intelligence; AI-QCT= artificial intelligence-guided quantitative computed tomography; CAC=coronary artery calcium; CMR=cardiac magnetic resonance; CNN=convolutional neural network; CT=computed tomography; CT-FFR=computed tomography fractional flow reserve; CTP=computed tomography perfusion; DL=deep learning; ESRGAN= Enhanced Super-Resolution Generative Adversarial Networks; ML=machine learning; MPI= myocardial perfusion imaging; MRA=magnetic resonance angiography; RNN-LSTM=recurrent neural network long short-term memory; SPECT=single-photon emission computed tomography.

Table S2. Quality assessment of diagnostic accuracy studies using QUADAS-2

Study	Risk of bias				Concerns regarding applicability		
	Patient selection	Index test	Reference standard	Flow and timing	Patient selection	Index test	Reference standard
Han 2020	Unclear	High	Low	Low	Low	Low	Low
Han 2023	Unclear	Low	Low	Low	Low	Low	Low
Han 2023	Low	Low	Low	Low	Low	Low	Low
Huang 2023	Low	Low	Low	Low	Low	Low	Low
Lin 2022	Unclear	Low	Low	Unclear	Low	Low	Low
Lipkin 2022	Low	Low	Low	Low	Low	Low	Low
Liu 2021	Unclear	High	Unclear	Low	Low	Low	Low
Xu 2022	Unclear	Low	Low	Low	Low	Low	Low
Xu 2021	Unclear	Low	Low	Low	Low	Low	Low

REFERENCES

1. Liu X, Mo X, Zhang H, Yang G, Shi C, Hau WK. A 2-Year Investigation of the Impact of the Computed Tomography-Derived Fractional Flow Reserve Calculated Using a Deep Learning Algorithm on Routine Decision-Making for Coronary Artery Disease Management. *Eur Radiol* (2021) 31(9):7039–46. Epub 20210225. doi: 10.1007/s00330-021-07771-7.

2. Sakellarios AI, Tachos N, Georga E, Rigas G, Kigka V, Siogkas P, et al., editors. A Novel Concept of the Management of Coronary Artery Disease Patients Based on Machine Learning Risk Stratification and Computational Biomechanics: Preliminary Results of Smartool Project. 2019; Singapore: Springer Nature Singapore.

3. Li Y, Qiu H, Hou Z, Zheng J, Li J, Yin Y, et al. Additional Value of Deep Learning Computed Tomographic Angiography-Based Fractional Flow Reserve in Detecting Coronary Stenosis and Predicting Outcomes. *Acta Radiol* (2022) 63(1):133–40. Epub 20210110. doi: 10.1177/0284185120983977.

4. Gohmann RF, Pawelka K, Seitz P, Majunke N, Heiser L, Renatus K, et al. Combined Ccta and Tavr Planning for Ruling out Significant Cad: Added Value of Ml-Based Ct-Ffr. *JACC Cardiovasc Imaging* (2022) 15(3):476–86. Epub 20211117. doi: 10.1016/j.jcmg.2021.09.013.

5. Ghorashi SM, Fazeli A, Hedayat B, Mokhtari H, Jalali A, Ahmadi P, et al. Comparison of Conventional Scoring Systems to Machine Learning Models for the Prediction of Major Adverse Cardiovascular Events in Patients Undergoing Coronary Computed Tomography Angiography. *Front Cardiovasc Med* (2022) 9:994483. Epub 20221026. doi: 10.3389/fcvm.2022.994483.

6. Tesche C, De Cecco CN, Baumann S, Renker M, McLaurin TW, Duguay TM, et al. Coronary Ct Angiography-Derived Fractional Flow Reserve: Machine Learning Algorithm Versus Computational Fluid Dynamics Modeling. *Radiology* (2018) 288(1):64–72. Epub 20180410. doi: 10.1148/radiol.2018171291.

7. von Knebel Doeberitz PL, De Cecco CN, Schoepf UJ, Duguay TM, Albrecht MH, van Assen M, et al. Coronary Ct Angiography-Derived Plaque Quantification with Artificial Intelligence Ct Fractional Flow Reserve for the Identification of Lesion-Specific Ischemia. *Eur Radiol* (2019) 29(5):2378–87. Epub 20181206. doi: 10.1007/s00330-018-5834-z.

8. Yu M, Lu Z, Li W, Wei M, Yan J, Zhang J. Ct Morphological Index Provides Incremental Value to Machine Learning Based Ct-Ffr for Predicting Hemodynamically Significant Coronary Stenosis. *Int J Cardiol* (2018) 265:256–61. doi: 10.1016/j.ijcard.2018.01.075.

9. Zreik M, van Hamersvelt RW, Khalili N, Wolterink JM, Voskuil M, Viergever MA, et al. Deep Learning Analysis of Coronary Arteries in Cardiac Ct Angiography for Detection of

Patients Requiring Invasive Coronary Angiography. *IEEE Trans Med Imaging* (2020) 39(5):1545–57. Epub 20191112. doi: 10.1109/TMI.2019.2953054.

10. van Hamersvelt RW, Zreik M, Voskuil M, Viergever MA, Isgum I, Leiner T. Deep Learning Analysis of Left Ventricular Myocardium in Ct Angiographic Intermediate-Degree Coronary Stenosis Improves the Diagnostic Accuracy for Identification of Functionally Significant Stenosis. *Eur Radiol* (2019) 29(5):2350–9. Epub 20181112. doi: 10.1007/s00330-018-5822-3.

11. Zreik M, Lessmann N, van Hamersvelt RW, Wolterink JM, Voskuil M, Viergever MA, et al. Deep Learning Analysis of the Myocardium in Coronary Ct Angiography for Identification of Patients with Functionally Significant Coronary Artery Stenosis. *Med Image Anal* (2018) 44:72–85. Epub 20171126. doi: 10.1016/j.media.2017.11.008.

12. Li Y, Yu M, Dai X, Lu Z, Shen C, Wang Y, et al. Detection of Hemodynamically Significant Coronary Stenosis: Ct Myocardial Perfusion Versus Machine Learning Ct Fractional Flow Reserve. *Radiology* (2019) 293(2):305–14. Epub 20190924. doi: 10.1148/radiol.2019190098.

13. Kumamaru KK, Fujimoto S, Otsuka Y, Kawasaki T, Kawaguchi Y, Kato E, et al. Diagnostic Accuracy of 3d Deep-Learning-Based Fully Automated Estimation of Patient-Level Minimum Fractional Flow Reserve from Coronary Computed Tomography Angiography. *Eur Heart J Cardiovasc Imaging* (2020) 21(4):437–45. doi: 10.1093/ehjci/jez160.

14. Coenen A, Kim YH, Kruk M, Tesche C, De Geer J, Kurata A, et al. Diagnostic Accuracy of a Machine-Learning Approach to Coronary Computed Tomographic Angiography-Based Fractional Flow Reserve: Result from the Machine Consortium. *Circ Cardiovasc Imaging* (2018) 11(6):e007217. doi: 10.1161/CIRCIMAGING.117.007217.

15. Zhang J, Xu K, Hu Y, Yang L, Leng X, Jin H, et al. Diagnostic Performance of Deep Learning and Computational Fluid Dynamics-Based Instantaneous Wave-Free Ratio Derived from Computed Tomography Angiography. *BMC Cardiovasc Disord* (2022) 22(1):33. Epub 20220205. doi: 10.1186/s12872-022-02469-0.

16. Tang CX, Wang YN, Zhou F, Schoepf UJ, Assen MV, Stroud RE, et al. Diagnostic Performance of Fractional Flow Reserve Derived from Coronary Ct Angiography for Detection of Lesion-Specific Ischemia: A Multi-Center Study and Meta-Analysis. *Eur J Radiol* (2019) 116:90–7. Epub 20190423. doi: 10.1016/j.ejrad.2019.04.011.

17. Hu X, Yang M, Han L, Du Y. Diagnostic Performance of Machine-Learning-Based Computed Fractional Flow Reserve (Ffr) Derived from Coronary Computed Tomography Angiography for the Assessment of Myocardial Ischemia Verified by Invasive Ffr. *Int J Cardiovasc Imaging* (2018) 34(12):1987–96. Epub 20180730. doi: 10.1007/s10554-018-1419-9.

18. Yu M, Dai X, Deng J, Lu Z, Shen C, Zhang J. Diagnostic Performance of Perivascular Fat Attenuation Index to Predict Hemodynamic Significance of Coronary Stenosis: A

Preliminary Coronary Computed Tomography Angiography Study. *Eur Radiol* (2020) 30(2):673–81. Epub 20190823. doi: 10.1007/s00330-019-06400-8.

19. Yang F, Shi K, Chen Y, Yin Y, Zhao Y, Zhang T. Effect of 320-Row Computed Tomography Acquisition Technology on Coronary Computed Tomography Angiography-Derived Fractional Flow Reserve Based on Machine Learning: Systolic and Diastolic Scan Acquisition. *J Comput Assist Tomogr* (2023) 47(2):205–11. Epub 20230307. doi: 10.1097/RCT.0000000000001423.

20. De Geer J, Coenen A, Kim YH, Kruk M, Tesche C, Schoepf UJ, et al. Effect of Tube Voltage on Diagnostic Performance of Fractional Flow Reserve Derived from Coronary Ct Angiography with Machine Learning: Results from the Machine Registry. *AJR Am J Roentgenol* (2019) 213(2):325–31. Epub 20190430. doi: 10.2214/AJR.18.20774.

21. Kawasaki T, Kidoh M, Kido T, Sueta D, Fujimoto S, Kumamaru KK, et al. Evaluation of Significant Coronary Artery Disease Based on Ct Fractional Flow Reserve and Plaque Characteristics Using Random Forest Analysis in Machine Learning. *Acad Radiol* (2020) 27(12):1700–8. Epub 20200210. doi: 10.1016/j.acra.2019.12.013.

22. Xue J, Li J, Sun D, Sheng L, Gong Y, Wang D, et al. Functional Evaluation of Intermediate Coronary Lesions with Integrated Computed Tomography Angiography and Invasive Angiography in Patients with Stable Coronary Artery Disease. *J Transl Int Med* (2022) 10(3):255–63. Epub 20220610. doi: 10.2478/jtim-2022-0018.

23. Pan Y, Zhu T, Wang Y, Deng Y, Guan H. Impact of Coronary Computed Tomography Angiography-Derived Fractional Flow Reserve Based on Deep Learning on Clinical Management. *Front Cardiovasc Med* (2023) 10:1036682. Epub 20230202. doi: 10.3389/fcvm.2023.1036682.

24. von Knebel Doeberitz PL, De Cecco CN, Schoepf UJ, Albrecht MH, van Assen M, De Santis D, et al. Impact of Coronary Computerized Tomography Angiography-Derived Plaque Quantification and Machine-Learning Computerized Tomography Fractional Flow Reserve on Adverse Cardiac Outcome. *Am J Cardiol* (2019) 124(9):1340–8. Epub 20190808. doi: 10.1016/j.amjcard.2019.07.061.

25. Qiao HY, Tang CX, Schoepf UJ, Tesche C, Bayer RR, 2nd, Giovagnoli DA, et al. Impact of Machine Learning-Based Coronary Computed Tomography Angiography Fractional Flow Reserve on Treatment Decisions and Clinical Outcomes in Patients with Suspected Coronary Artery Disease. *Eur Radiol* (2020) 30(11):5841–51. Epub 20200528. doi: 10.1007/s00330-020-06964-w.

26. Nous FMA, Budde RPJ, Lubbers MM, Yamasaki Y, Kardys I, Bruning TA, et al. Impact of Machine-Learning Ct-Derived Fractional Flow Reserve for the Diagnosis and Management of Coronary Artery Disease in the Randomized Crescent Trials. *Eur Radiol* (2020) 30(7):3692–701. Epub 20200312. doi: 10.1007/s00330-020-06778-w.

27. Renker M, Baumann S, Hamm CW, Tesche C, Kim WK, Savage RH, et al. Influence of Coronary Stenosis Location on Diagnostic Performance of Machine Learning-Based

Fractional Flow Reserve from Ct Angiography. *J Cardiovasc Comput Tomogr* (2021) 15(6):492–8. Epub 20210604. doi: 10.1016/j.jcct.2021.05.005.

28. Gao Z, Wang X, Sun S, Wu D, Bai J, Yin Y, et al. Learning Physical Properties in Complex Visual Scenes: An Intelligent Machine for Perceiving Blood Flow Dynamics from Static Ct Angiography Imaging. *Neural Netw* (2020) 123:82–93. Epub 20191130. doi: 10.1016/j.neunet.2019.11.017.

29. Feng Y, Xu Z, Zhang L, Zhang Y, Xu H, Zhuang X, et al. Machine-Learning-Derived Radiomics Signature of Pericoronary Tissue in Coronary Ct Angiography Associates with Functional Ischemia. *Front Physiol* (2022) 13:980996. Epub 20220926. doi: 10.3389/fphys.2022.980996.

30. Qiao HY, Tang CX, Schoepf UJ, Bayer RR, 2nd, Tesche C, Di Jiang M, et al. One-Year Outcomes of Ccta Alone Versus Machine Learning-Based Ffr(Ct) for Coronary Artery Disease: A Single-Center, Prospective Study. *Eur Radiol* (2022) 32(8):5179–88. Epub 20220217. doi: 10.1007/s00330-022-08604-x.

31. Yang J, Shan D, Wang X, Sun X, Shao M, Wang K, et al. On-Site Computed Tomography-Derived Fractional Flow Reserve to Guide Management of Patients with Stable Coronary Artery Disease: The Target Randomized Trial. *Circulation* (2023) 147(18):1369–81. Epub 20230304. doi: 10.1161/CIRCULATIONAHA.123.063996.

32. Kurata A, Fukuyama N, Hirai K, Kawaguchi N, Tanabe Y, Okayama H, et al. On-Site Computed Tomography-Derived Fractional Flow Reserve Using a Machine-Learning Algorithm - Clinical Effectiveness in a Retrospective Multicenter Cohort. *Circ J* (2019) 83(7):1563 – 71. Epub 20190608. doi: 10.1253/circj.CJ-19-0163.

33. Lee HJ, Kim YW, Kim JH, Lee YJ, Moon J, Jeong P, et al. Optimization of Ffr Prediction Algorithm for Gray Zone by Hemodynamic Features with Synthetic Model and Biometric Data. *Comput Methods Programs Biomed* (2022) 220:106827. Epub 20220421. doi: 10.1016/j.cmpb.2022.106827.

34. Qiao HY, Li JH, Schoepf UJ, Bayer RR, Tinnefeld FC, Di Jiang M, et al. Prognostic Implication of Ct-Ffr Based Functional Syntax Score in Patients with De Novo Three-Vessel Disease. *Eur Heart J Cardiovasc Imaging* (2020). Epub 20201113. doi: 10.1093/ehjci/jeaa256.

35. Ahmed AI, Han Y, Al Rifai M, Alnabelsi T, Nabi F, Chang SM, et al. Prognostic Value of Computed Tomography-Derived Fractional Flow Reserve Comparison with Myocardial Perfusion Imaging. *JACC Cardiovasc Imaging* (2022) 15(2):284–95. Epub 20211013. doi: 10.1016/j.jcmg.2021.09.007.

36. van Assen M, De Cecco CN, Eid M, von Knebel Doeberitz P, Scarabello M, Lavra F, et al. Prognostic Value of Ct Myocardial Perfusion Imaging and Ct-Derived Fractional Flow Reserve for Major Adverse Cardiac Events in Patients with Coronary Artery Disease. *J Cardiovasc Comput Tomogr* (2019) 13(3):26–33. Epub 20190212. doi: 10.1016/j.jcct.2019.02.005.

37. Brandt V, Bekeredjian R, Schoepf UJ, Varga-Szemes A, Emrich T, Aquino GJ, et al. Prognostic Value of Epicardial Adipose Tissue Volume in Combination with Coronary Plaque and Flow Assessment for the Prediction of Major Adverse Cardiac Events. *Eur J Radiol* (2022) 148:110157. Epub 20220115. doi: 10.1016/j.ejrad.2022.110157.

38. Al Rifai M, Ahmed AI, Han Y, Saad JM, Alnabelsi T, Nabi F, et al. Sex Differences in Machine Learning Computed Tomography-Derived Fractional Flow Reserve. *Sci Rep* (2022) 12(1):13861. Epub 20220816. doi: 10.1038/s41598-022-17875-9.

39. Lossnitzer D, Klenantz S, Andre F, Goerich J, Schoepf UJ, Pazzo KL, et al. Stable Patients with Suspected Myocardial Ischemia: Comparison of Machine-Learning Computed Tomography-Based Fractional Flow Reserve and Stress Perfusion Cardiovascular Magnetic Resonance Imaging to Detect Myocardial Ischemia. *BMC Cardiovasc Disord* (2022) 22(1):34. Epub 20220205. doi: 10.1186/s12872-022-02467-2.

40. Tang LM, Liu F, Dong TY, Yang F, Cui SJ. Synergistic Value of Fractional Flow Reserve and Low-Density Non-Calcified Plaque Based on Coronary Computed Tomography Angiography for the Identification of Lesion-Specific Ischemia. *Exp Ther Med* (2022) 24(5):701. Epub 20220929. doi: 10.3892/etm.2022.11637.

41. Yu M, Lu Z, Shen C, Yan J, Wang Y, Lu B, et al. The Best Predictor of Ischemic Coronary Stenosis: Subtended Myocardial Volume, Machine Learning-Based Ffr(Ct), or High-Risk Plaque Features? *Eur Radiol* (2019) 29(7):3647–57. Epub 20190322. doi: 10.1007/s00330-019-06139-2.

42. Di Jiang M, Zhang XL, Liu H, Tang CX, Li JH, Wang YN, et al. The Effect of Coronary Calcification on Diagnostic Performance of Machine Learning-Based Ct-Ffr: A Chinese Multicenter Study. *Eur Radiol* (2021) 31(3):1482–93. Epub 20200914. doi: 10.1007/s00330-020-07261-2.

43. Xu C, Xu M, Yan J, Li YY, Yi Y, Guo YB, et al. The Impact of Deep Learning Reconstruction on Image Quality and Coronary Ct Angiography-Derived Fractional Flow Reserve Values. *Eur Radiol* (2022) 32(11):7918–26. Epub 20220521. doi: 10.1007/s00330-022-08796-2.

44. Li S, Chen C, Qin L, Gu S, Zhang H, Yan F, et al. The Impact of Iterative Reconstruction Algorithms on Machine Learning-Based Coronary Ct Angiography-Derived Fractional Flow Reserve (Ct-Ffr(Ml)) Values. *Int J Cardiovasc Imaging* (2020) 36(6):1177–85. Epub 20200304. doi: 10.1007/s10554-020-01807-7.

45. Xu PP, Li JH, Zhou F, Jiang MD, Zhou CS, Lu MJ, et al. The Influence of Image Quality on Diagnostic Performance of a Machine Learning-Based Fractional Flow Reserve Derived from Coronary Ct Angiography. *Eur Radiol* (2020) 30(5):2525–34. Epub 20200131. doi: 10.1007/s00330-019-06571-4.

46. Martin SS, Mastrodicasa D, van Assen M, De Cecco CN, Bayer RR, Tesche C, et al. Value of Machine Learning-Based Coronary Ct Fractional Flow Reserve Applied to Triple-Rule-out Ct Angiography in Acute Chest Pain. *Radiol Cardiothorac Imaging* (2020) 2(3):e190137. Epub 20200625. doi: 10.1148/ryct.2020190137.

47. Yang F, Zhang Y, Lei P, Wang L, Miao Y, Xie H, et al. A Deep Learning Segmentation Approach in Free-Breathing Real-Time Cardiac Magnetic Resonance Imaging. *Biomed Res Int* (2019) 2019:5636423. Epub 20190730. doi: 10.1155/2019/5636423.

48. Morales MA, Assana S, Cai X, Chow K, Haji-Valizadeh H, Sai E, et al. An Inline Deep Learning Based Free-Breathing Ecg-Free Cine for Exercise Cardiovascular Magnetic Resonance. *J Cardiovasc Magn Reson* (2022) 24(1):47. Epub 20220811. doi: 10.1186/s12968-022-00879-9.

49. Zhang N, Yang G, Gao Z, Xu C, Zhang Y, Shi R, et al. Deep Learning for Diagnosis of Chronic Myocardial Infarction on Nonenhanced Cardiac Cine Mri. *Radiology* (2019) 291(3):606–17. Epub 20190430. doi: 10.1148/radiol.2019182304.

50. Masutani EM, Chandrupatla RS, Wang S, Zocchi C, Hahn LD, Horowitz M, et al. Deep Learning Synthetic Strain: Quantitative Assessment of Regional Myocardial Wall Motion at Mri. *Radiol Cardiothorac Imaging* (2023) 5(3):e220202. Epub 20230511. doi: 10.1148/ryct.220202.

51. Scannell CM, Veta M, Villa ADM, Sammut EC, Lee J, Breeuwer M, et al. Deep-Learning-Based Preprocessing for Quantitative Myocardial Perfusion Mri. *J Magn Reson Imaging* (2020) 51(6):1689–96. Epub 20191111. doi: 10.1002/jmri.26983.

52. Morales MA, Snel GJH, van den Boomen M, Borra RJH, van Deursen VM, Slart R, et al. Deepstrain Evidence of Asymptomatic Left Ventricular Diastolic and Systolic Dysfunction in Young Adults with Cardiac Risk Factors. *Front Cardiovasc Med* (2022) 9:831080. Epub 20220411. doi: 10.3389/fcvm.2022.831080.

53. Bottcher B, Beller E, Busse A, Cantre D, Yucel S, Oner A, et al. Fully Automated Quantification of Left Ventricular Volumes and Function in Cardiac Mri: Clinical Evaluation of a Deep Learning-Based Algorithm. *Int J Cardiovasc Imaging* (2020) 36(11):2239–47. Epub 20200716. doi: 10.1007/s10554-020-01935-0.

54. Zaidi HA, Jones RE, Hammersley DJ, Hatipoglu S, Balaban G, Mach L, et al. Machine Learning Analysis of Complex Late Gadolinium Enhancement Patterns to Improve Risk Prediction of Major Arrhythmic Events. *Front Cardiovasc Med* (2023) 10:1082778. Epub 20230207. doi: 10.3389/fcvm.2023.1082778.

55. Pezel T, Sanguineti F, Garot P, Unterseeh T, Champagne S, Toupin S, et al. Machine-Learning Score Using Stress Cmr for Death Prediction in Patients with Suspected or Known Cad. *JACC Cardiovasc Imaging* (2022) 15(11):1900–13. Epub 20220713. doi: 10.1016/j.jcmg.2022.05.007.

56. Pujadas ER, Raisi-Estabragh Z, Szabo L, McCracken C, Morcillo CI, Campello VM, et al. Prediction of Incident Cardiovascular Events Using Machine Learning and Cmr Radiomics. *Eur Radiol* (2023) 33(5):3488–500. Epub 20221213. doi: 10.1007/s00330-022-09323-z.

57. Yan Z, Su Y, Sun H, Yu H, Ma W, Chi H, et al. Segnet-Based Left Ventricular Mri Segmentation for the Diagnosis of Cardiac Hypertrophy and Myocardial Infarction. *Comput*

Methods Programs Biomed (2022) 227:107197. Epub 20221029. doi: 10.1016/j.cmpb.2022.107197.

58. Klug M, Shemesh J, Green M, Mayer A, Kerpel A, Konen E, et al. A Deep-Learning Method for the Denoising of Ultra-Low Dose Chest Ct in Coronary Artery Calcium Score Evaluation. *Clin Radiol* (2022) 77(7):e509–e17. Epub 20220409. doi: 10.1016/j.crad.2022.03.005.

59. Zeleznik R, Foldyna B, Eslami P, Weiss J, Alexander I, Taron J, et al. Deep Convolutional Neural Networks to Predict Cardiovascular Risk from Computed Tomography. *Nat Commun* (2021) 12(1):715. Epub 20210129. doi: 10.1038/s41467-021-20966-2.

60. Suh YJ, Kim C, Lee JG, Oh H, Kang H, Kim YH, et al. Fully Automatic Coronary Calcium Scoring in Non-Ecg-Gated Low-Dose Chest Ct: Comparison with Ecg-Gated Cardiac Ct. *Eur Radiol* (2023) 33(2):1254–65. Epub 20220913. doi: 10.1007/s00330-022-09117-3.

61. Fischer AM, Eid M, De Cecco CN, Gulsun MA, van Assen M, Nance JW, et al. Accuracy of an Artificial Intelligence Deep Learning Algorithm Implementing a Recurrent Neural Network with Long Short-Term Memory for the Automated Detection of Calcified Plaques from Coronary Computed Tomography Angiography. *J Thorac Imaging* (2020) 35 Suppl 1:S49–S57. doi: 10.1097/RTI.0000000000000491.

62. Peng AW, Dudum R, Jain SS, Maron DJ, Patel BN, Khandwala N, et al. Association of Coronary Artery Calcium Detected by Routine Ungated Ct Imaging with Cardiovascular Outcomes. *J Am Coll Cardiol* (2023) 82(12):1192–202. doi: 10.1016/j.jacc.2023.06.040.

63. Chamberlin J, Kocher MR, Waltz J, Snoddy M, Stringer NFC, Stephenson J, et al. Automated Detection of Lung Nodules and Coronary Artery Calcium Using Artificial Intelligence on Low-Dose Ct Scans for Lung Cancer Screening: Accuracy and Prognostic Value. *BMC Med* (2021) 19(1):55. Epub 20210304. doi: 10.1186/s12916-021-01928-3.

64. Yu J, Qian L, Sun W, Nie Z, Zheng D, Han P, et al. Automated Total and Vessel-Specific Coronary Artery Calcium (Cac) Quantification on Chest Ct: Direct Comparison with Cac Scoring on Non-Contrast Cardiac Ct. *BMC Med Imaging* (2022) 22(1):177. Epub 20221014. doi: 10.1186/s12880-022-00907-1.

65. van Assen M, Martin SS, Varga-Szemes A, Rapaka S, Cimen S, Sharma P, et al. Automatic Coronary Calcium Scoring in Chest Ct Using a Deep Neural Network in Direct Comparison with Non-Contrast Cardiac Ct: A Validation Study. *Eur J Radiol* (2021) 134:109428. Epub 20201121. doi: 10.1016/j.ejrad.2020.109428.

66. Mu D, Bai J, Chen W, Yu H, Liang J, Yin K, et al. Calcium Scoring at Coronary Ct Angiography Using Deep Learning. *Radiology* (2022) 302(2):309–16. Epub 20211123. doi: 10.1148/radiol.2021211483.

67. Wang W, Wang H, Chen Q, Zhou Z, Wang R, Wang H, et al. Coronary Artery Calcium Score Quantification Using a Deep-Learning Algorithm. *Clin Radiol* (2020) 75(3):237 e11–e16. Epub 20191111. doi: 10.1016/j.crad.2019.10.012.

68. van Velzen SGM, Lessmann N, Velthuis BK, Bank IEM, van den Bongard D, Leiner T, et al. Deep Learning for Automatic Calcium Scoring in Ct: Validation Using Multiple Cardiac Ct and Chest Ct Protocols. *Radiology* (2020) 295(1):66–79. Epub 20200211. doi: 10.1148/radiol.2020191621.

69. Winkel DJ, Suryanarayana VR, Ali AM, Gorich J, Buss SJ, Mendoza A, et al. Deep Learning for Vessel-Specific Coronary Artery Calcium Scoring: Validation on a Multi-Centre Dataset. *Eur Heart J Cardiovasc Imaging* (2022) 23(6):846–54. doi: 10.1093/ehjci/jeab119.

70. Lee JO, Park EA, Park D, Lee W. Deep Learning-Based Automated Quantification of Coronary Artery Calcification for Contrast-Enhanced Coronary Computed Tomographic Angiography. *J Cardiovasc Dev Dis* (2023) 10(4). Epub 20230328. doi: 10.3390/jcdd10040143.

71. Sandstedt M, Henriksson L, Janzon M, Nyberg G, Engvall J, De Geer J, et al. Evaluation of an Ai-Based, Automatic Coronary Artery Calcium Scoring Software. *Eur Radiol* (2020) 30(3):1671–8. Epub 20191114. doi: 10.1007/s00330-019-06489-x.

72. Lee JG, Kim H, Kang H, Koo HJ, Kang JW, Kim YH, et al. Fully Automatic Coronary Calcium Score Software Empowered by Artificial Intelligence Technology: Validation Study Using Three Ct Cohorts. *Korean J Radiol* (2021) 22(11):1764–76. Epub 20210726. doi: 10.3348/kjr.2021.0148.

73. Zhang N, Yang G, Zhang W, Wang W, Zhou Z, Zhang H, et al. Fully Automatic Framework for Comprehensive Coronary Artery Calcium Scores Analysis on Non-Contrast Cardiac-Gated Ct Scan: Total and Vessel-Specific Quantifications. *Eur J Radiol* (2021) 134:109420. Epub 20201124. doi: 10.1016/j.ejrad.2020.109420.

74. Inoue K, Seeman TE, Horwich T, Budoff MJ, Watson KE. Heterogeneity in the Association between the Presence of Coronary Artery Calcium and Cardiovascular Events: A Machine-Learning Approach in the Mesa Study. *Circulation* (2023) 147(2):132–41. Epub 20221031. doi: 10.1161/CIRCULATIONAHA.122.062626.

75. Barda N, Dagan N, Stemmer A, Yuval J, Bachmat E, Elnekave E, et al. Improving Cardiovascular Disease Prediction Using Automated Coronary Artery Calcium Scoring from Existing Chest Cts. *J Digit Imaging* (2022) 35(4):962–9. Epub 20220316. doi: 10.1007/s10278-021-00575-7.

76. Nakanishi R, Slomka PJ, Rios R, Betancur J, Blaha MJ, Nasir K, et al. Machine Learning Adds to Clinical and Cac Assessments in Predicting 10-Year Chd and Cvd Deaths. *JACC Cardiovasc Imaging* (2021) 14(3):615–25. Epub 20201028. doi: 10.1016/j.jcmg.2020.08.024.

77. Tamarappoo BK, Lin A, Commandeur F, McElhinney PA, Cadet S, Goeller M, et al. Machine Learning Integration of Circulating and Imaging Biomarkers for Explainable Patient-Specific Prediction of Cardiac Events: A Prospective Study. *Atherosclerosis* (2021) 318:76–82. Epub 20201113. doi: 10.1016/j.atherosclerosis.2020.11.008.

78. Al'Aref SJ, Maliakal G, Singh G, van Rosendael AR, Ma X, Xu Z, et al. Machine Learning of Clinical Variables and Coronary Artery Calcium Scoring for the Prediction of Obstructive Coronary Artery Disease on Coronary Computed Tomography Angiography: Analysis from the Confirm Registry. *Eur Heart J* (2020) 41(3):359–67. doi: 10.1093/eurheartj/ehz565.

79. Yu W, Yang L, Zhang F, Liu B, Shi Y, Wang J, et al. Machine Learning to Predict Hemodynamically Significant Cad Based on Traditional Risk Factors, Coronary Artery Calcium and Epicardial Fat Volume. *J Nucl Cardiol* (2023) 30(6):2593–606. Epub 20230711. doi: 10.1007/s12350-023-03333-0.

80. Commandeur F, Slomka PJ, Goeller M, Chen X, Cadet S, Razipour A, et al. Machine Learning to Predict the Long-Term Risk of Myocardial Infarction and Cardiac Death Based on Clinical Risk, Coronary Calcium, and Epicardial Adipose Tissue: A Prospective Study. *Cardiovasc Res* (2020) 116(14):2216–25. doi: 10.1093/cvr/cvz321.

81. Glowacki J, Krysinski M, Czaja-Ziolkowska M, Wasilewski J. Machine Learning-Based Algorithm Enables the Exclusion of Obstructive Coronary Artery Disease in the Patients Who Underwent Coronary Artery Calcium Scoring. *Acad Radiol* (2020) 27(10):1416–21. Epub 20191213. doi: 10.1016/j.acra.2019.11.016.

82. Cano-Espinosa C, Gonzalez G, Washko GR, Cazorla M, Jose Estepar RS. On the Relevance of the Loss Function in the Agatston Score Regression from Non-Ecg Gated Ct Scans. *Image Anal Mov Organ Breast Thorac Images (2018)* (2018) 11040:326–34. Epub 20180912. doi: 10.1007/978-3-030-00946-5_33.

83. Dobrolinska MM, Lazarenko SV, van der Zant FM, Does L, van der Werf N, Prakken NHJ, et al. Performance of Visual, Manual, and Automatic Coronary Calcium Scoring of Cardiac (13)N-Ammonia Pet/Low Dose Ct. *J Nucl Cardiol* (2023) 30(1):239–50. Epub 20220616. doi: 10.1007/s12350-022-03018-0.

84. Eslami P, Parmar C, Foldyna B, Scholtz JE, Ivanov A, Zeleznik R, et al. Radiomics of Coronary Artery Calcium in the Framingham Heart Study. *Radiol Cardiothorac Imaging* (2020) 2(1):e190119. Epub 20200227. doi: 10.1148/ryct.2020190119.

85. Huang Y, Ren Y, Yang H, Ding Y, Liu Y, Yang Y, et al. Using a Machine Learning-Based Risk Prediction Model to Analyze the Coronary Artery Calcification Score and Predict Coronary Heart Disease and Risk Assessment. *Comput Biol Med* (2022) 151(Pt B):106297. Epub 20221115. doi: 10.1016/j.compbiomed.2022.106297.

86. Lessmann N, van Ginneken B, Zreik M, de Jong PA, de Vos BD, Viergever MA, et al. Automatic Calcium Scoring in Low-Dose Chest Ct Using Deep Neural Networks with Dilated Convolutions. *IEEE Trans Med Imaging* (2018) 37(2):615–25. doi: 10.1109/TMI.2017.2769839.

87. Zreik M, van Hamersvelt RW, Wolterink JM, Leiner T, Viergever MA, Isgum I. A Recurrent Cnn for Automatic Detection and Classification of Coronary Artery Plaque and Stenosis in Coronary Ct Angiography. *IEEE Trans Med Imaging* (2019) 38(7):1588–98. Epub 20181128. doi: 10.1109/TMI.2018.2883807.

88. Penso M, Moccia S, Caiani EG, Caredda G, Lampus ML, Carerj ML, et al. A Token-Mixer Architecture for Cad-Rads Classification of Coronary Stenosis on Multiplanar Reconstruction Ct Images. *Comput Biol Med* (2023) 153:106484. Epub 20221226. doi: 10.1016/j.compbiomed.2022.106484.

89. Sun Z, Ng CKC. Artificial Intelligence (Enhanced Super-Resolution Generative Adversarial Network) for Calcium Deblooming in Coronary Computed Tomography Angiography: A Feasibility Study. *Diagnostics (Basel)* (2022) 12(4). Epub 20220414. doi: 10.3390/diagnostics12040991.

90. Han X, Luo N, Xu L, Cao J, Guo N, He Y, et al. Artificial Intelligence Stenosis Diagnosis in Coronary Cta: Effect on the Performance and Consistency of Readers with Less Cardiovascular Experience. *BMC Med Imaging* (2022) 22(1):28. Epub 20220217. doi: 10.1186/s12880-022-00756-y.

91. Kim Y, Choi AD, Telluri A, Lipkin I, Bradley AJ, Sidahmed A, et al. Atherosclerosis Imaging Quantitative Computed Tomography (Ai-Qct) to Guide Referral to Invasive Coronary Angiography in the Randomized Controlled Conserve Trial. *Clin Cardiol* (2023) 46(5):477–83. Epub 20230227. doi: 10.1002/clc.23995.

92. Yunus M, Sabarudin A, mohamed yusof akb, Hamid N, Abdul Karim MK. Automated Classification of Atherosclerosis in Coronary Computed Tomography Angiography Images Based on Radiomics Study Using Automatic Machine Learning. *Diagnostics (Basel)* (2022 Jul 8) 12(7):1660. doi: 10.1109/ICEARS53579.2022.9752423.

93. Li Y, Wu Y, He J, Jiang W, Wang J, Peng Y, et al. Automatic Coronary Artery Segmentation and Diagnosis of Stenosis by Deep Learning Based on Computed Tomographic Coronary Angiography. *Eur Radiol* (2022) 32(9):6037–45. Epub 20220408. doi: 10.1007/s00330-022-08761-z.

94. Jin X, Li Y, Yan F, Liu Y, Zhang X, Li T, et al. Automatic Coronary Plaque Detection, Classification, and Stenosis Grading Using Deep Learning and Radiomics on Computed Tomography Angiography Images: A Multi-Center Multi-Vendor Study. *Eur Radiol* (2022) 32(8):5276–86. Epub 20220315. doi: 10.1007/s00330-022-08664-z.

95. Huang Z, Xiao J, Wang X, Li Z, Guo N, Hu Y, et al. Clinical Evaluation of the Automatic Coronary Artery Disease Reporting and Data System (Cad-Rads) in Coronary Computed Tomography Angiography Using Convolutional Neural Networks. *Acad Radiol* (2023) 30(4):698–706. Epub 20220623. doi: 10.1016/j.acra.2022.05.015.

96. Huang Z, Yang Y, Wang Z, Hu Y, Cao B, Li M, et al. Comparison of Prognostic Value between Cad-Rads 1.0 and Cad-Rads 2.0 Evaluated by Convolutional Neural Networks Based Ccta. *Heliyon* (2023) 9(5):e15988. doi: https://doi.org/10.1016/j.heliyon.2023.e15988.

97. Lipkin I, Telluri A, Kim Y, Sidahmed A, Krepp JM, Choi BG, et al. Coronary Cta with Ai-Qct Interpretation: Comparison with Myocardial Perfusion Imaging for Detection of Obstructive Stenosis Using Invasive Angiography as Reference Standard. *AJR Am J Roentgenol* (2022) 219(3):407–19. Epub 20220420. doi: 10.2214/AJR.21.27289.

98. Han D, Liu J, Sun Z, Cui Y, He Y, Yang Z. Deep Learning Analysis in Coronary Computed Tomographic Angiography Imaging for the Assessment of Patients with Coronary Artery Stenosis. *Comput Methods Programs Biomed* (2020) 196:105651. Epub 20200709. doi: 10.1016/j.cmpb.2020.105651.

99. Liu CY, Tang CX, Zhang XL, Chen S, Xie Y, Zhang XY, et al. Deep Learning Powered Coronary Ct Angiography for Detecting Obstructive Coronary Artery Disease: The Effect of Reader Experience, Calcification and Image Quality. *Eur J Radiol* (2021) 142:109835. Epub 20210627. doi: 10.1016/j.ejrad.2021.109835.

100. Wu X, Deng L, Li W, Peng P, Yue X, Tang L, et al. Deep Learning-Based Acceleration of Compressed Sensing for Noncontrast-Enhanced Coronary Magnetic Resonance Angiography in Patients with Suspected Coronary Artery Disease. *J Magn Reson Imaging* (2023) 58(5):1521–30. Epub 20230227. doi: 10.1002/jmri.28653.

101. Lee H, Kang BG, Jo J, Park HE, Yoon S, Choi SY, et al. Deep Learning-Based Prediction for Significant Coronary Artery Stenosis on Coronary Computed Tomography Angiography in Asymptomatic Populations. *Front Cardiovasc Med* (2023) 10:1167468. Epub 20230621. doi: 10.3389/fcvm.2023.1167468.

102. Lin A, Manral N, McElhinney P, Killekar A, Matsumoto H, Kwiecinski J, et al. Deep Learning-Enabled Coronary Ct Angiography for Plaque and Stenosis Quantification and Cardiac Risk Prediction: An International Multicentre Study. *Lancet Digit Health* (2022) 4(4):e256–e65. doi: 10.1016/S2589-7500(22)00022-X.

103. Xu L, He Y, Luo N, Guo N, Hong M, Jia X, et al. Diagnostic Accuracy and Generalizability of a Deep Learning-Based Fully Automated Algorithm for Coronary Artery Stenosis Detection on Ccta: A Multi-Centre Registry Study. *Front Cardiovasc Med* (2021) 8:707508. Epub 20211105. doi: 10.3389/fcvm.2021.707508.

104. Xu J, Chen L, Wu X, Li C, Ai G, Liu Y, et al. Do Plaque-Related Factors Affect the Diagnostic Performance of an Artificial Intelligence Coronary-Assisted Diagnosis System? Comparison with Invasive Coronary Angiography. *Eur Radiol* (2022) 32(3):1866–78. Epub 20210926. doi: 10.1007/s00330-021-08299-6.

105. Matyas BB, Benedek I, Blindu E, Gerculy R, Rosca A, Rat N, et al. Elevated Fai Index of Pericoronary Inflammation on Coronary Ct Identifies Increased Risk of Coronary Plaque Vulnerability after Covid-19 Infection. *Int J Mol Sci* (2023) 24(8). Epub 20230417. doi: 10.3390/ijms24087398.

106. Fagman E, Alven J, Westerbergh J, Kitslaar P, Kercsik M, Cederlund K, et al. High-Quality Annotations for Deep Learning Enabled Plaque Analysis in Scapis Cardiac Computed Tomography Angiography. *Heliyon* (2023) 9(5):e16058. Epub 20230511. doi: 10.1016/j.heliyon.2023.e16058.

107. Li XN, Yin WH, Sun Y, Kang H, Luo J, Chen K, et al. Identification of Pathology-Confirmed Vulnerable Atherosclerotic Lesions by Coronary Computed Tomography Angiography Using Radiomics Analysis. *Eur Radiol* (2022) 32(6):4003–13. Epub 20220216. doi: 10.1007/s00330-021-08518-0.

108. Tesche C, Bauer MJ, Baquet M, Hedels B, Straube F, Hartl S, et al. Improved Long-Term Prognostic Value of Coronary Ct Angiography-Derived Plaque Measures and Clinical Parameters on Adverse Cardiac Outcome Using Machine Learning. *Eur Radiol* (2021) 31(1):486–93. Epub 20200728. doi: 10.1007/s00330-020-07083-2.

109. Dou G, Shan D, Wang K, Wang X, Liu Z, Zhang W, et al. Integrating Coronary Plaque Information from Ccta by Ml Predicts Mace in Patients with Suspected Cad. *J Pers Med* (2022) 12(4). Epub 20220407. doi: 10.3390/jpm12040596.

110. Jonas RA, Weerakoon S, Fisher R, Griffin WF, Kumar V, Rahban H, et al. Interobserver Variability among Expert Readers Quantifying Plaque Volume and Plaque Characteristics on Coronary Ct Angiography: A Clarify Trial Sub-Study. *Clin Imaging* (2022) 91:19–25. Epub 20220816. doi: 10.1016/j.clinimag.2022.08.005.

111. van Rosendael AR, Maliakal G, Kolli KK, Beecy A, Al'Aref SJ, Dwivedi A, et al. Maximization of the Usage of Coronary Cta Derived Plaque Information Using a Machine Learning Based Algorithm to Improve Risk Stratification; Insights from the Confirm Registry. *J Cardiovasc Comput Tomogr* (2018) 12(3):204–9. Epub 20180430. doi: 10.1016/j.jcct.2018.04.011.

112. Li N, Dong X, Zhu C, Shi K, Si N, Shi Z, et al. Model Development and Validation of Noninvasive Parameters Based on Coronary Computed Tomography Angiography to Predict Culprit Lesions in Acute Coronary Syndromes within 3 Years: Value of Plaque Characteristics, Hemodynamics and Pericoronary Adipose Tissue. *Quant Imaging Med Surg* (2023) 13(7):4325–38. Epub 20230510. doi: 10.21037/qims-22-1045.

113. Muscogiuri G, Chiesa M, Trotta M, Gatti M, Palmisano V, Dell'Aversana S, et al. Performance of a Deep Learning Algorithm for the Evaluation of Cad-Rads Classification with Ccta. *Atherosclerosis* (2020) 294:25–32. Epub 20191223. doi: 10.1016/j.atherosclerosis.2019.12.001.

114. Shu ZY, Cui SJ, Zhang YQ, Xu YY, Hung SC, Fu LP, et al. Predicting Chronic Myocardial Ischemia Using Ccta-Based Radiomics Machine Learning Nomogram. *J Nucl Cardiol* (2022) 29(1):262–74. Epub 20200618. doi: 10.1007/s12350-020-02204-2.

115. Benz DC, Ersozlu S, Mojon FLA, Messerli M, Mitulla AK, Ciancone D, et al. Radiation Dose Reduction with Deep-Learning Image Reconstruction for Coronary Computed Tomography Angiography. *Eur Radiol* (2022) 32(4):2620–8. Epub 20211118. doi: 10.1007/s00330-021-08367-x.

116. Lin A, Kolossvary M, Cadet S, McElhinney P, Goeller M, Han D, et al. Radiomics-Based Precision Phenotyping Identifies Unstable Coronary Plaques from Computed Tomography Angiography. *JACC Cardiovasc Imaging* (2022) 15(5):859–71. Epub 20220112. doi: 10.1016/j.jcmg.2021.11.016.

117. Jonas R, Patel T, Crabtree TR, Jennings RS, Heo R, Park HB, et al. Relation of Gender to Atherosclerotic Plaque Characteristics by Differing Angiographic Stenosis Severity. *Am J Cardiol* (2023) 204:276–83. Epub 20230808. doi: 10.1016/j.amjcard.2023.07.004.

118. Wang Y, Chen H, Sun T, Li A, Wang S, Zhang J, et al. Risk Predicting for Acute Coronary Syndrome Based on Machine Learning Model with Kinetic Plaque Features from Serial Coronary Computed Tomography Angiography. *Eur Heart J Cardiovasc Imaging* (2022) 23(6):800–10. doi: 10.1093/ehjci/jeab101.

119. Jonas RA, Barkovich E, Choi AD, Griffin WF, Riess J, Marques H, et al. The Effect of Scan and Patient Parameters on the Diagnostic Performance of Ai for Detecting Coronary Stenosis on Coronary Ct Angiography. *Clin Imaging* (2022) 84:149–58. Epub 20220203. doi: 10.1016/j.clinimag.2022.01.016.

120. Han X, He Y, Luo N, Zheng D, Hong M, Wang Z, et al. The Influence of Artificial Intelligence Assistance on the Diagnostic Performance of Ccta for Coronary Stenosis for Radiologists with Different Levels of Experience. *Acta Radiol* (2023) 64(2):496–507. Epub 20220407. doi: 10.1177/02841851221089263.

121. Han Q, Jing F, Sun Z, Liu F, Zhang J, Wang J, et al. Validation of the Commercial Coronary Computed Tomographic Angiography Artificial Intelligence for Coronary Artery Stenosis: A Cross-Sectional Study. *Quant Imaging Med Surg* (2023) 13(6):3789–801. Epub 20230412. doi: 10.21037/qims-22-1115.

122. Li L, Hu X, Tao X, Shi X, Zhou W, Hu H, et al. Radiomic Features of Plaques Derived from Coronary Ct Angiography to Identify Hemodynamically Significant Coronary Stenosis, Using Invasive Ffr as the Reference Standard. *Eur J Radiol* (2021) 140:109769. Epub 20210509. doi: 10.1016/j.ejrad.2021.109769.
